# Combined genetic deletion of GDF15 and FGF21 has modest effects on body weight, hepatic steatosis and insulin resistance in high fat fed mice

**DOI:** 10.1016/j.molmet.2022.101589

**Published:** 2022-09-02

**Authors:** Satish Patel, Afreen Haider, Anna Alvarez-Guaita, Guillaume Bidault, Julia Sarah El-Sayed Moustafa, Esther Guiu-Jurado, John A. Tadross, James Warner, James Harrison, Samuel Virtue, Fabio Scurria, Ilona Zvetkova, Matthias Blüher, Kerrin S. Small, Stephen O’Rahilly, David B. Savage

**Affiliations:** 1 University of Cambridge Metabolic Research Laboratories, Wellcome Trust-MRC Institute of Metabolic Science, Cambridge, CB2 0QQ, UK; 2 MRC Metabolic Diseases Unit, Wellcome-MRC Institute of Metabolic Science, University of Cambridge, Cambridge, UK; 3 Department of Medicine, Division of Cardiovascular Medicine, University of Cambridge, Cambridge, UK; 4 Medical Department III – Endocrinology, Nephrology, Rheumatology, University of Leipzig Medical Center, 04103 Leipzig, Germany; 5 Helmholtz Institute for Metabolic, Obesity and Vascular Research (HI-MAG) of the Helmholtz Zentrum München, University of Leipzig and University Hospital Leipzig, Leipzig, Germany; 6 Department of Twin Research and Genetic Epidemiology, King’s College London, St Thomas’ Campus, London, SE1 7EH, UK; 7 East Midlands and East of England Genomic Laboratory Hub & Department of Histopathology, Cambridge University Hospitals NHS Foundation Trust, Cambridge, UK

**Keywords:** GDF15, FGF21, Insulin resistance, Obesity

## Abstract

**Objectives:**

Obesity in humans and mice is associated with elevated levels of two hormones responsive to cellular stress, namely GDF15 and FGF21. Over-expression of each of these is associated with weight loss and beneficial metabolic changes but where they are secreted from and what they are required for physiologically in the context of overfeeding remains unclear.

**Methods:**

Here we used tissue selective knockout mouse models and human transcriptomics to determine the source of circulating GDF15 in obesity. We then generated and characterized the metabolic phenotypes of GDF15/FGF21 double knockout mice.

**Results:**

Circulating GDF15 and FGF21 are both largely derived from the liver, rather than adipose tissue or skeletal muscle, in obese states. Combined whole body deletion of FGF21 and GDF15 does not result in any additional weight gain in response to high fat feeding but it does result in significantly greater hepatic steatosis and insulin resistance than that seen in GDF15 single knockout mice.

**Conclusions:**

Collectively the data suggest that overfeeding activates a stress response in the liver which is the major source of systemic rises in GDF15 and FGF21. These hormones then activate pathways which reduce this metabolic stress.

## Highlights

• GDF15 knockout mice display greater obesity on a high fat diet.
• Hepatocytes are the major source of high fat diet-induced GDF15 and FGF21 in mice.
• GDF15 and FGF21 synergistically protect against obesity-induced hepatosteatosis.
• Deletion of GDF15 and FGF21 exacerbate insulin resistance in mice.

## 1 Introduction

Healthy adipose tissue is essential when coping with sustained nutritional overload and manifests a remarkable capacity to increase in size [1]. Depending on factors such as the duration and macronutrient source of excess caloric intake, fat mass can double in humans [2] and quadruple in rodent models [3]. Ultimately however, adipocytes start to die triggering a chronic inflammatory response, in which macrophages play a key role [[4], [5], [6], [7], [8], [9]]. Since excess energy cannot normally be excreted and humans, at least, manifest very small increases in energy expenditure when overfed [10], it necessarily accumulates in other ectopic sites (and in plasma lipoproteins to some extent), setting off a cascade of metabolic complications. In the liver, this ectopic lipid accumulation is strongly associated with the development of insulin resistance [[11], [12], [13], [14], [15], [16], [17]] and in some cases a hepatic inflammatory response. As many as a third of patients with non-alcoholic fatty liver disease (NAFLD) progress to cirrhosis and some of these go on to develop hepatocellular carcinoma [18,19]. Lipid also accumulates in skeletal muscle [15,20,21] where it has again been linked to the pathogenesis of insulin resistance and type 2 diabetes [13,22]. Although there are some reports of ‘inflammation’ in skeletal muscle in this context, the underlying mechanisms, and the impact on muscle itself and on systemic insulin resistance are less clear [23].

Ectopic lipid accumulation in the liver has been reported by several groups to be associated with activation of the integrated stress response (ISR) [[24], [25], [26], [27]], whereas in skeletal muscle of humans or high fat fed mice there is limited evidence of induction of the ISR [24,28,29]. The ISR is triggered by a variety of stressors (such as endoplasmic reticulum stress, mitochondrial stress, hypoxia and, amino acid or glucose depletion) which activate one or more of at least four kinases leading to the phosphorylation of eukaryotic initiation factor 2 alpha (eIF2α) [30]. This in turn attenuates overall protein synthesis whilst permitting selective translation of specific proteins required for cellular adaptation, repair and alteration of metabolic homeostasis [31,32]. We [33] and others [[34], [35], [36]] have shown that activation of the ISR (via nutritional, genetic or pharmacological stressors) is associated with increased expression of growth differentiation factor 15 (GDF15) and fibroblast growth factor 21 (FGF21). Plasma levels of both FGF21 [[37], [38], [39], [40], [41]] and GDF15 [[42], [43], [44]] are known to be increased in obese humans and rodents as well as in other metabolic disease states such as insulin resistance [45,46], NAFLD [[47], [48], [49], [50], [51], [52]] and mitochondrial disease [53,54]. Both stress-induced cytokines, GDF15 and FGF21, have attracted considerable interest as potential therapies for obesity and its associated metabolic disease [55].

FGF21 was discovered in 2000 and reported to be primarily expressed in the liver [56] though it is now known to be more widely expressed, including other key metabolic tissues such as adipose tissue, skeletal muscle and pancreas [57,58]. FGF21 was found to be a potent regulator of glucose uptake in an *in vitro* screen in 3T3-L1 adipocytes [41] and has subsequently been associated with a range of pleiotropic effects, including improved insulin sensitivity and β cell function, reduced hepatic lipogenesis, and increased energy expenditure via brown fat thermogenesis [59]. Using tissue-specific knockout mice, plasma FGF21 was shown to be primarily derived from the liver in response to high fat feeding [60]; it then acts centrally to regulate energy expenditure and body weight [61,62]. FGF21 is also reported to act in an auto/paracrine fashion when secreted by adipocytes and pancreatic exocrine cells [[63], 64, [65], [66]]. Molecularly, FGF21 signals via the FGF receptor-1c and its cognate co-receptor β-klotho which is predominantly expressed in target tissue such as the CNS and adipocytes [[67], [68], [69]]. Both transgenic overexpression and exogenous administration of supraphysiologic levels of FGF21 in genetic- or diet-induced obese (DIO) rodents, substantially reduces body weight, hypertriglyceridaemia and hyperglycaemia [41,70,71]. These responses were shown to be associated with reductions in hepatic and intramyocellular lipid content (TAG and DAG) [72]. Beneficial metabolic effects of exogenous FGF21 administration were also observed in obese or diabetic primates [73,74] and in humans [74,75], although the improvements in hyperglycaemia were disappointingly modest in humans. FGF21 deficiency in mice has been linked with reduced insulin sensitivity in a high fat diet setting [76], but the impact on body weight remains unclear as studies have reported both lower and higher body weight in FGF21 null mice [76,77]. Some studies suggested that the weight of FGF21 knockout mice was increased early after transition to a high fat diet but that this difference was subsequently lost [66,78].

GDF15, originally identified as a gene upregulated in activated macrophages [79], can be expressed in almost all cell types, and is relatively highly expressed in several tissues including the liver, kidneys, intestines and especially the placenta. Plasma GDF15 is elevated in a range of human diseases, in addition to obesity and the metabolic syndrome, where it is widely considered to be a useful biomarker [[80], [81], [82]]. GDF15 mRNA expression is elevated within the liver and adipose tissue of high fat fed mice [33]. GDF15 null mice weigh more than WT littermates on a high fat diet and are glucose intolerant [83]. Meanwhile, GDF15 over-expressing transgenic mice are protected from diet-induced obesity and display improved insulin sensitivity [84]. Concordantly, pharmacological treatment with recombinant GDF15 reduces food intake and body weight in obese rodents and primates [43,85,86]. These metabolic impacts of GDF15 are mediated via the GFRAL receptor along with its tyrosine kinase coreceptor Ret in the hindbrain [87,88].

Here we sought firstly to clarify the principal source of GDF15 in HFD fed mice. Having shown that the liver is a major source of GDF15 in this context, similarly to what has previously been reported for FGF21, we proceeded to evaluate the phenotypic impact of deleting both genes in mice. As both are present at least as far back as zebrafish [89,90] and both have been implicated in weight loss, we hypothesized that they might act synergistically to alleviate the stress imposed by nutritional overload so we generated and characterised GDF15:FGF21 double knockout mice.

## 2 Materials and methods

### 2.1 Animal husbandry

Mice were maintained in ventilated cages with group housing (2–5 per cage), unless specified otherwise for indirect calorimetry and food intake experiments, on a 12 h light/12 h dark cycle (lights on 06:00–18:00), in a temperature-controlled (20–24 °C) facility, with *ad libitum* access to food and water. During the experimental protocol, all mice were fed either *ad libitum* or fasted as stated prior to some tests. All animal studies were performed on male mice and carried out at two facilities at the University of Cambridge, UK. This research was regulated under the Animals (Scientific Procedures) Act 1986 Amendment Regulations 2012 following ethical review by the University of Cambridge Animal Welfare and Ethical Review Body (AWERB).

### 2.2 Mouse models

Mice carrying the GDF15 knockout-first “tm1a” allele [C57BL/6N-Gdf15^tm1a(KOMP)Wtsi/H^] were obtained through the IMPC, from the Harwell production centre (https://www.mousephenotype.org/data/alleles/MGI:1346047/tm1a%2528KOMP%2529Wtsi). A “conditional ready GDF15 Tm1c” allele [C57BL/6N-Gdf15^tm1c(KOMP)Wtsi^/H] expressing mouse was generated in house. Briefly, one-cell stage embryos (obtained from super-ovulated wild type C57Bl/6N females fertilised *in vitro* with sperm from homozygous GDF15 Tm1a male) were injected into the pronucleus with 100ng/ul StemMACS Flp Recombinase mRNA (Miltenyi Biotec) then transferred into the uteri of pseudo pregnant recipient females (F1 hybrids from C57Bl/6J female × CBA/Ca male crosses). Mice from the transfer (F1 mice) were analysed for the presence of the GDF15 Tm1c and Tm1a alleles. The F1 founders were crossed twice with wild type C657Bl/6N mice before establishing the GDF15 Tm1c and GDF15 Tm1a colonies. The GDF15 Tm1c mouse model contains loxP sites flanking exon 2 of GDF15 gene (see https://www.mousephenotype.org/data/alleles/MGI:1346047/tm1c(KOMP)Wtsi). FGF21^−/−^ mice (B6N; 129S5-Fgf21tm1Lex/Mmucd) were generated on a C57BL/6N background using sperm obtained from MMRRC (https://www.mmrrc.org/catalog/sds.php?mmrrc_id=32306). GDF15/FGF21 dKO mice and wild-type littermates were obtained from het × het breeding set-ups with all strains maintained on a C57BL6/N background. Genotyping to confirm derivation of all mouse lines was done by PCR using the primers described in the key resources table.

Myeloid-specific deletions of GDF15 were generated using two separate strategies:

(1) GDF15 ^fl/fl^ (GDF15 Tm1c) mice were bred to transgenic *Lyz2*^Cre/+^ mice carrying Cre recombinase under the control of the LysM promoter (kindly provided by T.Vidal-Puig, Institute of Metabolic Sciences, Univ of Cambridge). This line was maintained on a mixed C57BL6N/J background. Genotypes were confirmed by PCR for the presence of Cre and for the detection of the floxed GDF15 allele. GDF15^fl/fl^ and GDF15 ^fl/fl^ LysM-Cre were used in this study.
(2) 24 × C57BL/6N wild type mice (4–6 weeks of age) were purchased from Charles River, Italy and were allowed to acclimatize for 1 week before being irradiated. Mice received two separate doses of 5.5 Gy of radiation using Caesium 60 source with a 4 h gap in between doses. 1–2 h post-irradiation, donor bone marrow cells (10 million/mouse) from either male GDF15 KO Tm1a or wild type littermates were injected into the tail veins of the irradiated mice. The mice were then housed under standard conditions for one month, monitored and weighed regularly until 12 weeks of age prior to experimentation.

Hepatocyte-specific GDF15 KO mice were generated by breeding GDF15 ^fl/fl^ (GDF15 Tm1c) to *Alb*^Cre/+^ mice carrying Cre recombinase under the control of the albumin promoter (kindly provided by A. Kaser, Univ of Cambridge). This line was maintained on a C57BL/6N background and genotypes were confirmed by PCR for the presence of Cre as well as floxed GDF15 allele. GDF15^fl/fl^ and GDF15 ^fl/fl^ Alb-cre were used in this study.

### 2.3 Mouse studies

Starting at the age of 5–6 weeks, mice were fed either a control chow (R105-25, Safe Diets) or a 60% high fat diet (D12492i, Research Diets) for a period of 14–26 weeks. For all cohorts, the mice were weighed weekly and body composition was determined every 4 weeks by Time-Domain Nuclear Magnetic Resonance (TD-NMR) using a Minispec Live Mice Analyzer (LF50, Bruker). Tail blood samples were collected into heparinized micro blood tubes (01605-00, Hawksley), centrifuged at 13,000 × *g* for 4 min and plasma was collected for the analysis of hormones or lipids as indicated in figures.

Food intake studies were performed with singly housed mice over a 2-week period where both body and food weight were recorded.

Indirect calorimetry analyses on the GDF15 liver KO mice were performed on single-housed HFD fed mice in Promethion cages (Sable Systems International) for 48 h according to manufacturer’s standard protocols. Carbon dioxide (CO_2_) and oxygen (O_2_) concentrations and the incoming air supply were determined every 3 min for each chamber/mouse. Analysis of the WT, FGF21 KO, GDF15KO and dKO animals was performed using an in-house calorimetry system. Carbon dioxide (CO_2_) and oxygen (O_2_) concentrations and the incoming air supply were determined every 11 min for each chamber/mouse. Energy expenditure was calculated using the modified Weir equation [EE J/min = 15.818 × VO_2_ (ml/min) + 5.176 × VCO_2_ (ml/min)] and converted to kcal/h using the conversion factor 1 J = 0.239 cal.

The mice were euthanized at the end of the experiment with tissues harvested, weighed and frozen at −80 °C or fixed in 10% neutral buffered formalin (NBF).

### 2.4 Glucose and insulin tolerance test

Glucose and insulin tolerance tests (GTT and ITT) were conducted on mice that were fasted for 6 h (08:00–14:00) in clean cages. Mice were injected intraperitoneally with 1 g/kg glucose (for GTT) or 1.5 U/kg insulin (for ITT) and blood glucose (tail vein) was measured at the indicated times using a AlphaTRAK 2 m. Blood samples (∼30 μl) were collected during a GTT for insulin measurements. Homeostatic model assessment for insulin resistance (HOMA-IR) was calculated as 6 h fasting glucose (mmol/l) × 6 h fasting insulin (ng/ml)/22.5.

### 2.5 Serum, plasma and media analysis

Tail blood samples from mice were collected for plasma analysis. Mouse leptin and insulin were measured using a single-plex MesoScale Discovery assay kit (Rockville, MD, USA product code K152BYC-2 for leptin and K152BZC-3 for insulin). The assay was performed according to the manufacturer’s instructions using calibrators provided by MSD. Mouse GDF15 was measured using a DuoSet ELISA (R&D Systems product code DY6385) which had been modified to run as an electrochemiluminescence assay on the MesoScale Discovery assay platform. Mouse FGF21 was analysed using a Quantikine ELISA kit (R&D Systems, product code MF2100) following the manufacturer’s instructions. ALT, AST, Cholesterol and triglycerides were measured on the Siemens Dimension EXL autoanalyser using Siemens reagents and calibrators. Human GDF15 was also measured using a DuoSet ELISA (R&D Systems product code DY957) that had been converted to MSD format. Comprehensive Quality Control procedures were followed for all measurements and no results were reported without passing QC checks.

Mouse sample measurements were performed by the Cambridge MRC MDU Mouse Biochemistry Laboratory and human assays were provided by the NIHR Cambridge BRC Core Biochemical Assay laboratory (CBAL).

### 2.6 Histology, immunohistochemistry and tissue architecture analysis

Tissues were dissected and placed into 10% neutral buffered formalin for 48 h at room temperature, transferred to 70% ethanol and embedded into paraffin. 5 μM sections were cut using a Leica microtome, mounted onto Superfrost Plus slides (Thermo Fisher Scientific) and stained for hematoxylin and eosin. Detection of mouse *Gdf15* and *Fgf21* mRNA was performed on formalin-fixed paraffin-embedded sections, obtained from 45% high fat diet fed, wild-type C57BL6/J mice [33], using Advanced Cell Diagnostics (ACD) RNAscope 2.5 LS Reagent Kit-RED (no. 322150) and RNAscope LS 2.5 Probe Mm-Gdf15-O1 (no. 442948) (ACD) and a RNAscope LS 2.5 Probe Mm-Fgf21-C2 (no. 460938-C2). Slides were processed as previously described [91]. Positive and negative controls were run in parallel each time

All slides were imaged using the Zeiss AxioScan.Z1 Slide Scanner at × 20 (standard) or × 40 magnification (RNAscope). For the RNAscope slides, three Z-stacks spanning a total of 2 μm were acquired and merged into a single extended depth of focus (EDF) image with maximum projection processing, and then sharpened using Unsharp Masking (ZEN Blue, Zeiss). Detection of mouse F4/80 was conducted on formalin-fixed paraffin-embedded sections using a F4/80 rat anti-mouse monoclonal antibody (BioRad MCA497) for 1 h at room temperature on a Leica BOND RX Automated Stainer. Antigen retrieval was performed for 20 min at 98 °C at a low pH (pH6.0, citrate based BOND Epitope Retrieval Solution) before incubation with the F4/80 primary antibody and followed by a rabbit anti-rat IgG secondary antibody (Bethyl A110-322A) incubation at a dilution of 1:250 for 1 h at room temperature. DAB was used to detect the immunostaining and slides were counterstained with haematoxylin.

Hepatic lipid droplet area was determined using the vacuole detection algorithm of the Halo software (Indica labs). Hepatic lipid area was expressed as a percentage of total hepatic tissue area scanned per section.

### 2.7 Hepatic lipid content measurement

Accumulation of hepatic lipid was determined using a modified Folch method. In brief, 25 mg of frozen liver was homogenized in 1.2 ml of a 2:1 ratio choloroform:methanol mixture (Sigma-Aldrich, Dorset, UK). Deionized water (240 μl) was then added to the mix, vortexed thoroughly, and samples centrifuged for 10 min at 16,100 × *g* to generate a distinct organic and aqueous phase. The lower organic phase (500 μl) was collected into a pre-weighed glass tube and dried under a nitrogen stream with the lipid content weighed and normalised to total liver weight.

### 2.8 Isolation of primary adipose stromal fractions

Epididymal adipose tissues were removed at the time of sacrifice and minced finely into small pieces and resuspended in 5 ml Hanks’ Balanced Salt Solution (HBSS, H9269, Sigma), 0.1 g bovine serum albumin (BSA, A8806, Sigma), and 10 mg collagenase type II (C6885, Sigma). The tissue was completely disaggregated by incubation in a 37 °C shaker for approximately 15 min. The digested material was topped with 5 ml of ice-cold MACS buffer (2 mM EDTA, 0.5% BSA in PBS) and allowed to settle for 5 min at room temperature. The adipocyte fraction floating on top was collected by pipetting, washed twice with MACS buffer and then snap frozen in Buffer RLT (Qiagen) for RNA extraction. The remainder of the digested solution (approximately 9 ml) was filtered through a 100 μm nylon mesh cell strainer (Falcon 352360) and centrifuged at 400 × *g* for 5 min. The pellet containing the stromalvascular fraction (SVF) was collected and washed once with MACS buffer and then resuspended with CD11b microbeads (130-049-601-Millteyni Biotec) in MACS buffer. The CD11b positive and negative cells (SVF) were separated and collected using MACS LS columns (130-042-401, Miltenyi Biotec) that were placed onto a magnetic field of a MACS separator (Miltenyi Biotec). The cells were centrifuged at 400 × *g* for 5 min and snap frozen in Buffer RLT (Qiagen) for RNA extraction.

### 2.9 Culture and differentiation of bone-marrow derived macrophages

Femur and tibia bones from mice were isolated, cleaned and flushed with 10 ml of RPMI-1640 media (Sigma) through each bone using a 25G syringe. The flushed bone marrow cells were passed into a 100 μm cell strainer and centrifuged at 400 × *g* for 5 min and resuspended in macrophage differentiation medium [RPMI-1640 with 20% L929 conditioned medium, 10% heat-inactivated FBS and 100 U/ml penicillin-streptomycin (Thermo Fisher Scientific)]. Total bone-marrow cells were counted using a Countess II automated cell counter (Thermo Fisher) and seeded onto 10 cm non-culture treated plates (Falcon) at a density of 5 × 10^6^ cells per plate and maintained for 7 days at 37 °C in a humidified atmosphere of 5% CO_2_. On day 5 of differentiation, medium was removed, and 10 ml of fresh macrophage differentiation medium was added to each plate. On day 7 of differentiation, macrophages were detached using ice-cold PBS containing 1 mM EDTA and centrifuged at 400 g for 5 min. The cells were resuspended in macrophage differentiation medium and seeded onto 24 well plate for 24 h prior to experiments.

To make L929 conditioned medium, L929 cells (CCL-1, ATCC) were seeded in DMEM supplemented with 10% heat-inactivated FBS, 100 U/ml penicillin-streptomycin and 2 mM l-glutamine (Sigma) at a density of 250,000 cells per 50 ml of medium per T175 tissue culture flask. Medium was harvested after 1 week of culture, and then 50 ml of fresh DMEM supplemented with 10% heat-inactivated FBS, 100 U/ml penicillin-streptomycin and 2 mM l-glutamine was added onto cells and harvested 1 week later. Batches obtained after the first and second weeks of culture were mixed at a 1:1 ratio, aliquoted and stored at −20 °C.

### 2.10 RNA isolation, cDNA synthesis and qPCR for mouse studies

Following treatments, cells were lysed with Buffer RLT (Qiagen) containing 1% 2-Mercaptoethanol and processed through a Qiashredder with total RNA extracted using the RNeasy isolation kit according to manufacturer’s instructions (Qiagen). Meanwhile for mice, tissues were harvested and immediately snap frozen in liquid nitrogen and stored at −80 °C until further analysis. For RNA isolation, approximately 30–50 mg of tissue was placed in Lysing Matrix D tubes and homogenized in 800 μl TRI reagent (Sigma-Aldrich) using the Fastprep-24 Homogenizer for 30 s at 4–6 m/s (MP Biomedical). The resultant supernatant was transferred to an RNAse free tube and 200 μl chloroform (Sigma) added. The samples were vortexed and centrifuged at 13,000 rpm for 15 min at 4 °C. The upper phase was then transferred to a RNAse free tube and mixed with equal volume of 70% ethanol before loading onto RNA isolation spin columns (Qiagen). RNA was then extracted using the RNeasy isolation kit following the manufacturer’s instructions.

RNA concentration and quality was determined by Nanodrop. 400–600 ng of total RNA was treated with DNAase I (Promega) and then converted to cDNA using Lunascript (NEB). Quantitative RT-PCR was carried out with either TaqMan™ Universal PCR Master Mix or SYBR Green PCR master mix on the QuantStudio 7 Flex Real time PCR system (Applied Biosystems). All reactions were carried out in either duplicate or triplicate and Ct values were obtained. Relative differences in the gene expression were normalized to expression levels of housekeeping genes B2M, 36b4 or RPL13a (geometrical mean) using the standard curve method. Primer sequences are shown in the key resources table.

### 2.11 Human studies

For the Twins cohort, details of the study are described in Buil et al., 2015 [92]. Briefly, the study included 856 female individuals of European ancestry recruited from the TwinsUK Adult twin registry. The study was approved by The St. Thomas' Research Ethics Committee (REC). Volunteers gave informed consent and signed an approved consent form before the biopsy procedure.

For the obese cohort, 525 metabolically well-characterized participants of the Leipzig Obesity BioBank were recruited at four bariatric surgery centers in Leipzig, Karlsruhe, Dresden and Gera (all in Germany) (Table 1). All subjects underwent clinical phenotyping as described previously [[93], [94], [95]]. All subjects had a stable weight, defined as no fluctuations of >2% of body weight for at least 3 months before surgery. According to American Diabetes Association (ADA) criteria [96] 205 study participants (∼39%) were diagnosed with T2D. We defined the following exclusion criteria: (i) thyroid dysfunction, (ii) alcohol or drug abuse, (iii) pregnancy and (iv) treatment with thiazolidinediones. The study was approved by the ethics committee of the University of Leipzig (Approval numbers: 159-12-21052012 and 017-12-23012012). The study designs followed the Declaration of Helsinki and all participants gave written informed consent prior to participation.

**Table:** Key resources table

Reagent Type (species) or resource	Designation	Source or reference	Identifiers	Additional information
Genetic reagent (*M. musculus*)	C57BL/6N-Gdf15^tm1a(KOMP)Wtsi^/H	MRC Harwell	RRID:IMSR_HAR:5024	Also known as Gdf15 KO or Gdf15^−/−^ mouse
Genetic reagent (*M. musculus*)	B6N; 129S5-Fgf21tm1Lex/Mmucd	MMRRC	RRID:MMRRC_032306-UCD	Also known as Fgf21 KO or Fgf21^−/−^ mouse
Genetic reagent (*M. musculus*)	B6N: Gdf15/Fgf21	This study		Also known as Gdf15^−/−^/Fgf21 ^−/−^or dKO mouse
Genetic reagent (*M. musculus*)	C57BL/6N-Gdf15^tm1c(KOMP)Wtsi^/H	This study	RRID:IMSR_EM:10460	Also known as Gdf15 floxed mouse
Genetic reagent (*M. musculus*)	*B6.129P2-Lyz2*^*tm1(cre)Ifo*^*/J*	Clausen BE et al., 1999	RRID:IMSR_JAX:004781	Also known as *LysM* Cre mouse. Donated by T.Vidal Puig, IMS, Uni of Camb
Genetic reagent (*M. musculus*)	*B6.Cg-Speer6-ps1*^*Tg(Alb-cre)21Mgn*^*/J*	Postic et al., 1999	RRID:IMSR_JAX:003574	Also *known as Alb* Cre mouse. Donated by A. Kaser, Uni of Camb
Chemical compound, drug	Formalin solution neutral buffered 10%	Sigma-Aldrich	Cat# HT501128	
Chemical compound, drug	Haematoxylin (Mayer)	Pioneer Research Chemicals	Cat# PRC/R/42	
Chemical compound, drug	Eosin (1٪ aqueous)	Pioneer Research Chemicals	Cat# PRC/66/1	
Chemical compound, drug	Paramat Gurr Paraffin Wax	VWR	Cat# 361147B	
Chemical compound, drug	Xylene	Thermo Fisher Scientific	Cat# 12632916	
Commercial assay or kit	dNTPs	Promega	Cat# U151B	
Commercial assay or kit	RNeasy Mini Kit	Qiagen	Cat# 74106	For mouse studies
Commercial assay or kit	RNeasy Lipid Tissue Mini Kit	Qiagen	Cat# 74804	For obese human study
Commercial assay or kit	Qiashredder	Qiagen	Cat# 79656	
Commercial assay or kit	RQ1 RNase-Free DNase	Promega	Cat# M6101	
Commercial assay or kit	Lunascript RT SuperMix Kit	New England Biolabs	Cat# E3010L	
Commercial assay or kit	TaqMan™ Universal PCR Master Mix	Thermo Fisher Scientific	Cat# 4304437	
Commercial assay or kit	SYBR™ Green PCR Master Mix	Thermo Fisher Scientific	Cat# 4309155	
Commercial assay or kit	Mouse Insulin Assay	MesoScale Discovery	Cat# K152BZC-3	
Commercial assay or kit	Mouse Leptin Assay	MesoScale Discovery	Cat# K152BYC-2	
Commercial assay or kit	Mouse GDF15 DuoSet ELISA	R&D Systems	Cat# DY6385	Modified (see methods)
Commercial assay or kit	Mouse FGF21 Quantikine ELISA	R&D Systems	Cat# MF2100	
Commercial assay or kit	Human GDF15 DuoSet ELISA	R&D Systems	Cat# DY957	Modified (see methods)
Commercial assay or kit	Cholesterol	Siemens Healthcare	Cat#DF27	
Commercial assay or kit	ALT	Siemens Healthcare	Cat#DF143	
Commercial assay or kit	AST	Siemens Healthcare	Cat#DF41A	
Commercial assay or kit	Triglyceride Assay	Siemens Healthcare	Cat# DF69A	
Commercial assay or kit	Miltenyi Biotec, Inc. CD11B MICROBEADS	Miltenyi Biotec	Cat# 130-049-601	
Commercial assay or kit	Miltenyi Biotec, Inc. LS COLUMNS	Miltenyi Biotec	Cat# 130-042-401	
Commercial assay or kit	MidiMACS™ Separator and MACS MultiStand	Miltenyi Biotec	Cat# 130-042-302	
			Cat# 130-042-303	
Commercial assay or kit	RNAscope® 2.5 LS Probe -Mm-Fgf21-C2	Advanced Cell Diagnostics (ACD)	Cat# 442948	
Commercial assay or kit	RNAscope® LS 2.5 Probe Mm-Gdf15-O1	Advanced Cell Diagnostics (ACD)	Cat# 442948	
Commercial assay or kit	RNAscope® 2.5 LS Reagent Kit-RED	Advanced Cell Diagnostics (ACD)	Cat# 322150	
Commercial assay or kit	Bond Polymer Refine Red Detection Kit	Leica Biosystems	Cat# DS9390	
Commercial assay or kit	Mouse *Gdf15* Taqman assay	Thermo Fisher Scientific	Cat# Mm00442228_m1	
Commercial assay or kit	Mouse *Fgf21* Taqman assay	Thermo Fisher Scientific	Cat# Mm00840165_g1	
Commercial assay or kit	Mouse *Perilipin1* Taqman assay	Thermo Fisher Scientific	Cat#Mm00558672_m1	
Commercial assay or kit	Mouse *Acaca* Taqman assay	Thermo Fisher Scientific	Cat# Mm01304257_m1	
Commercial assay or kit	Mouse *Pparα* Taqman assay	Thermo Fisher Scientific	Cat# Mm00440939_m1	
Commercial assay or kit	Mouse *Acox1* Taqman assay	Thermo Fisher Scientific	Cat# Mm00443579_m1	
Commercial assay or kit	Mouse *Cd36* Taqman assay	Thermo Fisher Scientific	Cat# Mm01135198_m1	
Commercial assay or kit	Mouse *Acadl* Taqman assay	Thermo Fisher Scientific	Cat# Mm00599660_m1	
Commercial assay or kit	Human *Gdf15* Taqman assay	Thermo Fisher Scientific	Cat# Hs00171132_m1	
Commercial assay or kit	Human *CD68* Taqman assay	Thermo Fisher Scientific	Cat# Hs02836816_g1	
Commercial assay or kit	Human *GAPDH* Taqman assay	Thermo Fisher Scientific	Cat# Hs 02786624_g1	
Other	60% High Fat Diet	Research Diets	Cat# D12492i	HFD for mice
Other	Rats, Mice& Hamsters unique diet	Safe Diets	Cat# R105-25	Control chow diet for mice
Other	Heparinized capillary tubes	Hawksley	Cat# 1605-00	For mouse blood collection
Other	Dulbecco’s Minimum Essential Medium (DMEM)	Sigma-Aldrich	Cat# D6546	
Other	RPM1 1640 Medium	Sigma-Aldrich	Cat# R8758	
Other	l-Glutamine Solution 200 mM	Sigma-Aldrich	Cat# 9202C	
Other	D-PBS	Sigma-Aldrich	Cat# D8537	
Other	Hanks’ Balanced Salt Solution	Sigma-Aldrich	Cat# H9269	
Other	Fetal Bovine Serum	PAN-Biotech	Cat# P30-3602	
Other	Newborn Calf Serum	Sigma-Aldrich	Cat# N4637-500M	
Other	Bovine Serum Albumin	Sigma-Aldrich	Cat# A6003	
Other	2 ml Screw Top Vials (Amber) and Screw Caps	Agilent Technologies	Cat# 8010-0543	For lipid isolation
Other	EcoMount Mounting Medium	Biocare Medical	Cat# EM897L	
Other	Epitope Retrieval Solution 2	Leica Biosystems	Cat# AR9640	
Other	Nanodrop 2000	Thermo Fisher Scientific	NA	For DNA/RNA estimation
Other	Countess II	Thermo Fisher Scientific	NA	Cell counter
Other	FastPrep-24	MP Biomedical	Cat# 116004500	For cell/tissue homogenization
Other	Lysing Matrix D, 2 ml Tube	MP Biomedical	Cat# 116913100	For cell/tissue homogenization
Other	Sterile Cell strainer (100 μm nylon mesh)	Fisherbrand	Cat# 22363549	For tissue homogenization
Other	AlphaTrack2 Glucometer	Abbot Laboratories	Cat# CFMU305-H0201	For blood glucose measurement
Other	AlphaTrack2 strips	Zoetis	Cat# 71681-01	For blood glucose measurement
Other	Minispec LF series (TD-NMR)	Bruker	Cat# LF50	For animal body composition analysis
Other	Microtome	Leica	Cat# RM2255	For histological sectioning
Other	Axioscan Z1 slide scanner	Zeiss	NA	For histological imaging
Other	HistoStar embedding workstation	Thermo Fisher Scientific	NA	For histological slide preparation
Antibody	F480	Biorad	MCA497	For IHC on FFPE sections
Peptide, recombinant protein	Insulin (Actrapid)	Novo Nordisk	Cat# 041-7642	
Peptide, recombinant protein	Collagenase Type II from *Clostridium histolyticum*	Sigma-Aldrich	Cat# C6885	
Sequence-based reagent	Mouse GDF15_WT Forward GGGCAATCCTTCTGCCTCCA	In this study	NA	Genotyping primers for Gdf15 KO mice (617bp product for WT allele; 943 bp product for Tm1a allele)
	Mouse GDF15_WT Reverse GCACGCTTCAGGGGCCTAGT			
	Mouse GDF15_Tm1a Reverse CGCCCAAGGCCATACAAGTG			
Sequence-based reagent	Mouse GDF15_tm1c Forward CTGGGAAGACAGGTGTAGGC	In this study	NA	Genotyping primers for Gdf15 floxed mice (780bp for Tm1c allele and 586bp for WT allele)
	Mouse GDF15_tm1c ReverseGCACGCTTCAGGGGCCTAGT			
Sequence-based reagent	Neo Forward CCTGTCATCTCACCTTGCTCCT	In this study	NA	Genotyping primers for Fgf21 KO mice (669bp product for Tm1Lex allele and 370bp product for WT allele)
	*Fgf21_Exon3* Forward AAGGACTCCCCAAACCAGG			
	DNA480-2 ReverseTGACAGGGTCTCAGGTTCAA			
Sequence-based reagent	Cre forward CCTGGAAAATGCTTCTGTCCG	In this study	NA	Genotyping primers for LysM mice and Alb Cre Mice (375bp product)
	Cre Reverse CAGGGTGTTATAAGCAATCCC			
Sequence-based reagent	Mouse *CHOP* Forward CCACCACACCTGAAAGCAGAA	In this study	NA	QRT-PCR primer
	Mouse *CHOP* Reverse AGGTGAAAGGCAGGGACTCA			
Sequence-based reagent	Mouse *Atf4* Forward GGGTTCTGTCTTCCACTCCA	In this study	NA	QRT-PCR primer
	Mouse *Atf4* Reverse AAGCAGCAGAGTCAGGCTTTC			
Sequence-based reagent	Mouse *Atf3* Forward TGGAGATGTCAGTCACCAAGTCT	In this study	NA	QRT-PCR primer
	Mouse *Atf3* Reverse GCAGCAGCAATTTTATTTCTTTCT			
Sequence-based reagent	Mouse *Il1b* Forward CTGGTGTGTGACGTTCCCATTA	In this study	NA	QRT-PCR primer
	Mouse *Il1b* Reverse CCGACAGCACGAGGCTTT			
Sequence-based reagent	Mouse *Mcp1* Forward CCACTCACCTGCTGCTACTCA	In this study	NA	QRT-PCR primer
	Mouse *Mcp1* Reverse TGGTGATCCTCTTGTAGCTCTCC			
Sequence-based reagent	Mouse *Fasn* Forward CCTGGATAGCATTCCGAACCT	In this study	NA	QRT-PCR primer
	Mouse *Fasn* Reverse AGCACATCTCGAAGGCTACACA			
Sequence-based reagent	Mouse *Lpl* Forward TGGAGAAGCCATCCGTGTG	In this study	NA	QRT-PCR primer
	Mouse *Lpl* Reverse TCATGCGAGCACTTCACCAG			
Sequence-based reagent	Mouse *Dgat2* Forward TTCTGCACAGACTGTGGCTGATA	In this study	NA	QRT-PCR primer
	Mouse *Dgat2* Reverse TGGTCAGCAGGTTGTGTGTCTTCA			
Sequence-based reagent	Mouse *Tnfa* Forward CATCTTCTCAAAATTCGAGTGACAA	In this study	NA	QRT-PCR primer
	Mouse *Tnfa* Reverse TGGGAGTAGACAAGGTACAACCC			
Sequence-based reagent	Mouse *Cpt1a* Forward CCTGGGCATGATTGCAAAG	In this study	NA	QRT-PCR primer
	Mouse *Cpt1a* Reverse GCCACTCACGATGTTCTTCGT			
Sequence-based reagent	Mouse *Scd1* Forward CGTCTGGAGGAACATCATTC	In this study	NA	QRT-PCR primer
	Mouse *Scd1* Reverse AGCGCTGGTCATGTAGTA			
Sequence-based reagent	Mouse *Elovl6* Forward TGCAGGAAAACTGGAAGAAGTCT	In this study	NA	QRT-PCR primer
	Mouse *Elovl6* Reverse ATGCCGACCACCAAAGATAAA			
Sequence-based reagent	Mouse *Srebp1c* Forward GGCACTAAGTGCCCTCAACCT	In this study	NA	QRT-PCR primer
	Mouse *Srebp1c* Reverse GCCACATAGATCTCTGCCAGTGT			
Sequence-based reagent	Mouse *F4/80* Forward CAGATACAGCAATGCCAAGCA	In this study	NA	QRT-PCR primer
	Mouse *F4/80* Reverse GATTGTGAAGGTAGCATTCACAAGTG			
Sequence-based reagent	Mouse *B2m* Forward ACTGATACATACGCCTGCAGAGTT	In this study	NA	QRT-PCR primer
	Mouse *B2m* Reverse TCACATGTCTCGATCCCAGTAGA			
Sequence-based reagent	Mouse 36b4 Forward AGATGCAGCAGATCCGCAT	In this study	NA	QRT-PCR primer
	Mouse 36b4 Reverse GTTCTTGCCCATCAGCACC			
Sequence-based reagent	Mouse *Rpl13* Forward GGATCCCTCCACCCTATGACA	In this study	NA	QRT-PCR primer
	Mouse *Rpl13* Reverse CTGGTACTTCCACCCGACCTC			
Software, algorithm	GraphPad PRISM 9.3.1 (350)	1992–2021 GraphPad Software, LLC	RRID:SCR_002798	For graphical representation and mouse studies statistical analysis
Software, algorithm	HALO	Indica Labs	NA	For histological image analysis
Software, algorithm	STAR 2.4.0.1	NA	https://github.com/alexdobin/STAR/releases	
			https://github.com/alexdobin/STAR	
Software, algorithm	QTLtools	NA	https://qtltools.github.io/qtltools/	
Software, algorithm	Gencode version 19	NA	https://www.gencodegenes.org/human/release_19.html	
Software, algorithm	R package 3.5.1	NA	(https://www.r-project.org/)	For TwinsUK human study statistical analysis
Software, algorithm	SPSS/PC + Version 27.0	SPSS, Chicago, IL, USA		For obese human study statistical analysis
Software, algorithm	CIBERSORT	NA	https://cibersort.stanford.edu/	
Software, algorithm	QuantStudio 7 Flex Real time PCR system	Thermo Fisher Scientific	NA	For mouse studies
Software, algorithm	QuantStudio 6 Flex Fast Real time PCR system	Thermo Fisher Scientific	NA	For Obese human study
Software, algorithm	JASP (version 0.16.3)		https://jasp-stats.org/	For energy expenditure ANCOVA analysis

**Table 1:** Anthropometric and metabolic characterization of the cohort.

	BMI <30 kg/m^2^ (n = 16)	BMI 30–40 kg/m^2^ (n = 51)	BMI >40 kg/m^2^ (n = 458)
Age (years)	65.09 ± 13.06	48.31 ± 11.66	47.02 ± 11.86
Men/Women (n)	12/4	12/39	119/339
T2D (n)	3	20	182
Body weight (kg)	78.88 ± 10.84	109.23 ± 12.67	143.29 ± 26.78
Height (m)	1.76 ± 0.09	1.70 ± 0.07	1.69 ± 0.09
BMI (kg/m^2^)	25.43 ± 2.31	37.51 ± 2.57	50.01 ± 7.30
Body fat (%)	22.88 ± 2.88	44.05 ± 9.76	49.59 ± 9.84
Waist circumference (cm)	95.17 ± 11.82	122.50 ± 9.19	145.56 ± 160.3
Hip circumference (cm)	98.33 ± 8.37	124.00 ± 11.31	148.06 ± 14.03
WHR	0.97 ± 0.09	0.99 ± 0.17	0.99 ± 0.10
FPG (mmol/l)	5.73 ± 0.62	5.98 ± 1.83	6.47 ± 2.45
FPI (pmol/l)	22.17 ± 11.72	111.86 ± 86.35	148.30 ± 110.35
HbA1c (%)	5.72 ± 0.48	6.05 ± 1.33	6.08 ± 1.16
HOMA-Index	0.77 ± 0.47	4.85 ± 4.24	6.07 ± 6.39
Total cholesterol (mmol/l)	5.01 ± 0.76	5.20 ± 1.81	4.85 ± 1.09
HDL-cholesterol (mmol/l)	1.21 ± 0.29	1.25 ± 0.30	1.17 ± 0.66
LDL-cholesterol (mmol/l)	2.97 ± 0.37	3.33 ± 1.03	3.07 ± 0.95
Triglycerides (mmol/l)	1.23 ± 0.50	1.86 ± 1.21	2.12 ± 2.42
CrP (mg/L)	8.76 ± 12.60	7.70 ± 15.54	13.16 ± 18.03

Data are given as means ± SD. AT, adipose tissue; BMI, body max index; CrP, C-reactive protein; FPG, fasting plasma glucose; FPI, fasting plasma insulin; HDL-C, high density lipoprotein cholesterol; LDL-C, low density lipoprotein cholesterol; SAT, subcutaneous adipose tissue; VAT, visceral adipose tissue; WHR, waist to hip ratio.

### 2.12 Gene expression data for human studies

In the TwinsUK cohort, gene expression levels in subcutaneous adipose tissue were measured by RNA-Seq in 765 female twins, as previously described [92]. Briefly, punch biopsies were collected from a sun-protected area of the abdomen from each participant, from which adipose tissue was separated and RNA extracted. Gene expression was measured by RNA-Seq, with RNA-Seq reads aligned to the hg19 reference genome using STAR [97] version 2.4.0.1, as fully described elsewhere [98]. Gene level counts were generated using the quan function of QTLtools [99] and Gencode version 19 [100], and rank-based inverse normal transformation then applied to gene counts per million (CPMs) prior to all downstream analyses. Gene expression levels were then adjusted for RNA-Seq technical covariates using linear mixed effects models, with expression levels of each gene in turn treated as a dependent variable, with median insert size and mean GC content included as fixed effects, and date of RNA sequencing and RNA-Seq primer index as random effects.

In the obese cohort, paired samples of abdominal omental AT (visceral, VAT) and subcutaneous AT (SAT) were obtained from 525 Caucasian men (n = 143) and women (n = 382), who underwent open abdominal surgery as described previously [94,95]. The age ranged from 18 to 80 years and body mass index (BMI) from 19 to 60 kg/m^2^. AT was immediately frozen in liquid nitrogen and stored at −80 °C. RNA was extracted from AT by using the RNeasy Lipid Tissue Mini Kit (Qiagen, Hilden, Germany), and qPCR was performed as described elsewhere [101,102]. Real-time quantitative PCR was performed with the TaqMan Assay predesigned by Applied Biosystems (Foster City, CA, USA) for the detection of human *GDF15* (Hs00171132_m1), *CD68* (Hs02836816_g1) and *GAPDH* (Hs 02786624_g1) mRNA expression in AT. All reactions were carried out in 96-well plates using the QuantStudio (TM) 6 Flex System Fast Real-Time PCR system. *GDF1*5 mRNA expression was calculated relative to *GAPDH* mRNA expression

### 2.13 Estimation of adipose tissue cell type proportions

Adipose tissue cell type proportions were estimated from RNA-Seq data in TwinsUK using CIBERSORT [103], as reported previously elsewhere [98]. Estimated cell types included in our analysis were adipocytes, microvascular endothelial cells (MVEC) and macrophages.

### 2.14 Quantification and statistical analysis

Cell and mouse quantitative data are reported as mean ± standard deviation (SD). As indicated in the figure legends, differences between means were assessed by two-tailed Student’s *t* tests or One-way ANOVA or Two-way ANOVA with Sidak/Tukey multiple comparisons test using either GraphPad Prism 9 software (GraphPad, San Diego) or with SAS version 9.4, Cary, N. Carolina. Statistical significance was defined as *p* < 0.05. Metabolic rate was determined using ANCOVA with average energy expenditure over 48 h as the dependent variable, average body weight over 48 h as a covariate and genotype as a fixed factor. ANCOVA analysis was performed using JASP (version 0.16.3)

Association analyses in TwinsUK cohort: Association of adipose tissue *GDF15* expression levels was assessed to *GDF15* serum levels, adipose tissue expression levels of macrophage markers *CD68* and *EMR1*, and adipose tissue estimated macrophage proportions, using linear mixed effects models. Participants with type 2 diabetes (n = 32) were excluded from all analyses. Linear mixed effects models were fitted using the lmer function from the lme4 package [104] in R [105] version 3.5.1.

Rank-based inverse normal transformation was applied to serum GDF15 serum levels. GDF15 serum levels were treated as a dependent variable, with adipose tissue *GDF15* gene expression residuals, adjusted for technical covariates as previously described, as a predictor variable. Covariates included BMI and age as fixed effects, and family and zygosity as random effects. Family and zygosity are both random effects that permit identification of the family a twin belongs to, and their clonality (MZ/DZ status).

To assess association of adipose tissue *GDF15* expression levels with gene expression levels of macrophage markers *CD68* and *EMR1*, and adipose tissue estimated macrophage proportion, GDF15 gene expression residuals were treated as a dependent variable, and each of adipose tissue expression residuals of *CD68*, and *EMR1*, and adipose tissue estimated macrophage proportion, were used in turn as predictor variables. Models included the same fixed and random effect covariates as described for the GDF15 serum level model. For each analysis, the full model was then compared to a null model where the trait of interest was omitted, using a 1 degree of freedom ANOVA.

For obese human subject data, prior to statistical analysis, non-normally distributed parameters were logarithmically (ln) transformed to approximate a normal distribution. Results are expressed as mean ± SD. Linear regression analysis were used to assess the relationships between *GDF1*5 mRNA expression and phenotypic traits. Pearson´s correlation analyses were conducted using two-way bivariate correlations. Differences in *GDF1*5 mRNA expression between visceral and subcutaneous AT were assessed using the paired Student’s t-test or one-way ANOVA. Statistical analyses were performed using SPSS/PC + for Windows statistical package (version 27.0; SPSS, Chicago, IL, USA).

## 3 Results

### 3.1 Impact of GDF15 deletion on body weight regulation in mice

**Figure 1:**
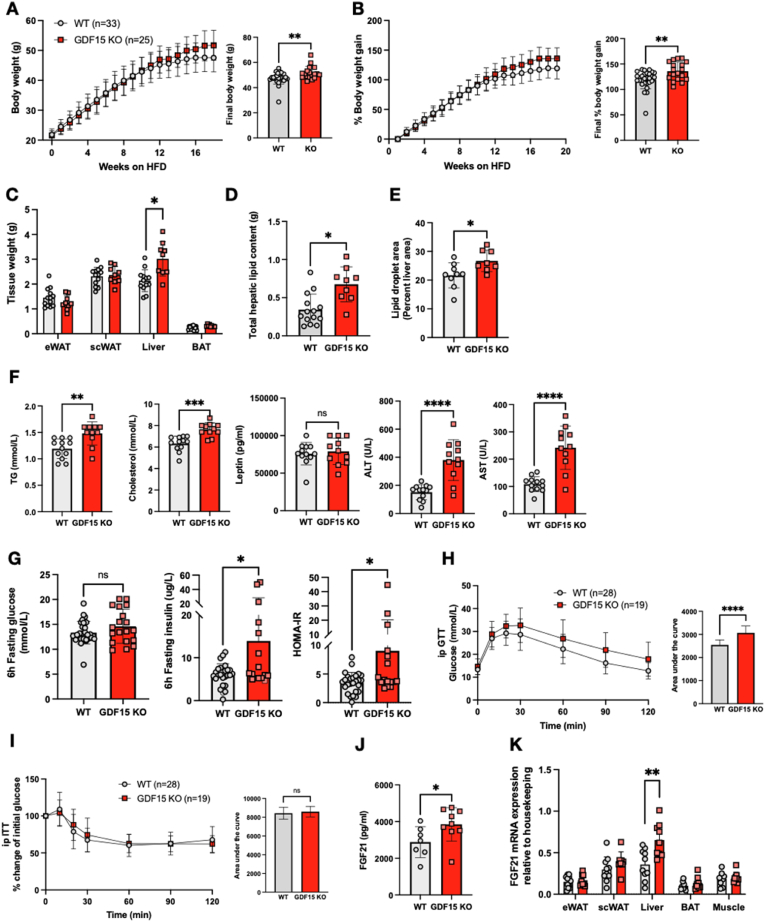
**Phenotypic characterization of GDF15 knockout mice on a highfat diet (HFD)**. (A and B) Body weight and percent body weight gain of wild type (WT) and GDF15 KO mice fed a 60% HFD; Inset, final body weight and percent body weight gain. (C) Weight of epididymal white adipose tissue (eWAT), subcutaneous white adipose tissue (scWAT), liver and brown adipose tissue (BAT), harvested at the end of the study, 25 weeks of HFD (n = 13,9). (D) Weight of total hepatic lipids in g; total lipid extracted from 25 mg tissue was normalized to total liver weight (n = 14,9). (E) Lipid droplet area (Percent liver area) determined from histological analyses of haematoxylin/eosin (H&E) stained liver sections (n = 9,8). (F) Plasma triglycerides (TG), cholesterol, leptin, alanine transaminase (ALT) and aspartate transaminase (AST) from random fed mice after 16 weeks of HFD-feeding (n = 12,11). (G) Blood glucose, plasma insulin and HOMA-IR levels from 6 h fasted mice, after 16 weeks of HFD feeding (n = 16–29). (H and I) Blood glucose levels during intraperitoneal (ip) glucose tolerance test (GTT) and percent change from initial blood glucose levels during insulin tolerance test (ITT) after 16 weeks of HFD feeding. Inset, area under the curve analysis of glucose over time. (J) Plasma FGF21 levels from random fed mice at 16 weeks of HFD feeding (n = 7,9). (K) FGF21 mRNA expression in tissues from WT and GDF15 KO mice after 25 weeks HFD feeding (n = 8–11). All data are means ± S.D ∗/∗∗/∗∗∗/∗∗∗∗ - p < 0.05/0.01/0.001/0.0001.

In order to confirm prior reports suggesting that GDF15 null mice are heavier when fed a high fat diet (HFD) [83], wild-type (WT) and GDF15 KO mice were fed a 60% HFD for ∼20 weeks. The GDF15 KO mice displayed a small but significant increase in absolute body weight compared to their WT littermates (Figure 1A), the difference becoming evident from about 10 weeks into the HFD, particularly when the data was displayed as percent body weight gain (Figure 1B). Daily food intake was similarly very modestly increased in the GDF15 null mice whereas energy expenditure was similar to that of WT mice (Supplemental Figure 1A and B). Subcutaneous, epididymal and brown adipose tissue weights were similar between the groups, but GDF15 KO mice manifested increased liver tissue mass (Figure 1C). Biochemical (Figure 1D) and histological analyses revealed elevated hepatic lipid content in GDF15 KO mice (Figure 1E and Supplemental Figure 1C) and plasma triglycerides; cholesterol, leptin and liver enzyme concentrations were also higher than those of wildtype littermates (Figure 1F). Blood glucose concentrations were similar in 6 h fasted mice whereas plasma insulin and HOMA-IR were elevated suggesting mild insulin resistance (Figure 1G). Glucose tolerance was also mildly impaired in the GDF15 KO group whereas insulin tolerance was similar to that of WT mice suggesting that the insulin resistance might primarily relate to the liver rather than peripheral tissues (Figure 1H,I). High fat feeding is known to increase plasma FGF21 in mice [33] and was even higher in GDF15 KO mice after 16 weeks of HFD feeding (Figure 1J). In keeping with this observation, FGF21 mRNA was increased in the liver of the GDF15 KOs but not in WAT, BAT or skeletal muscle (Figure 1K).

### 3.2 GDF15 is upregulated in adipose tissue macrophages and hepatocytes

**Figure 2:**
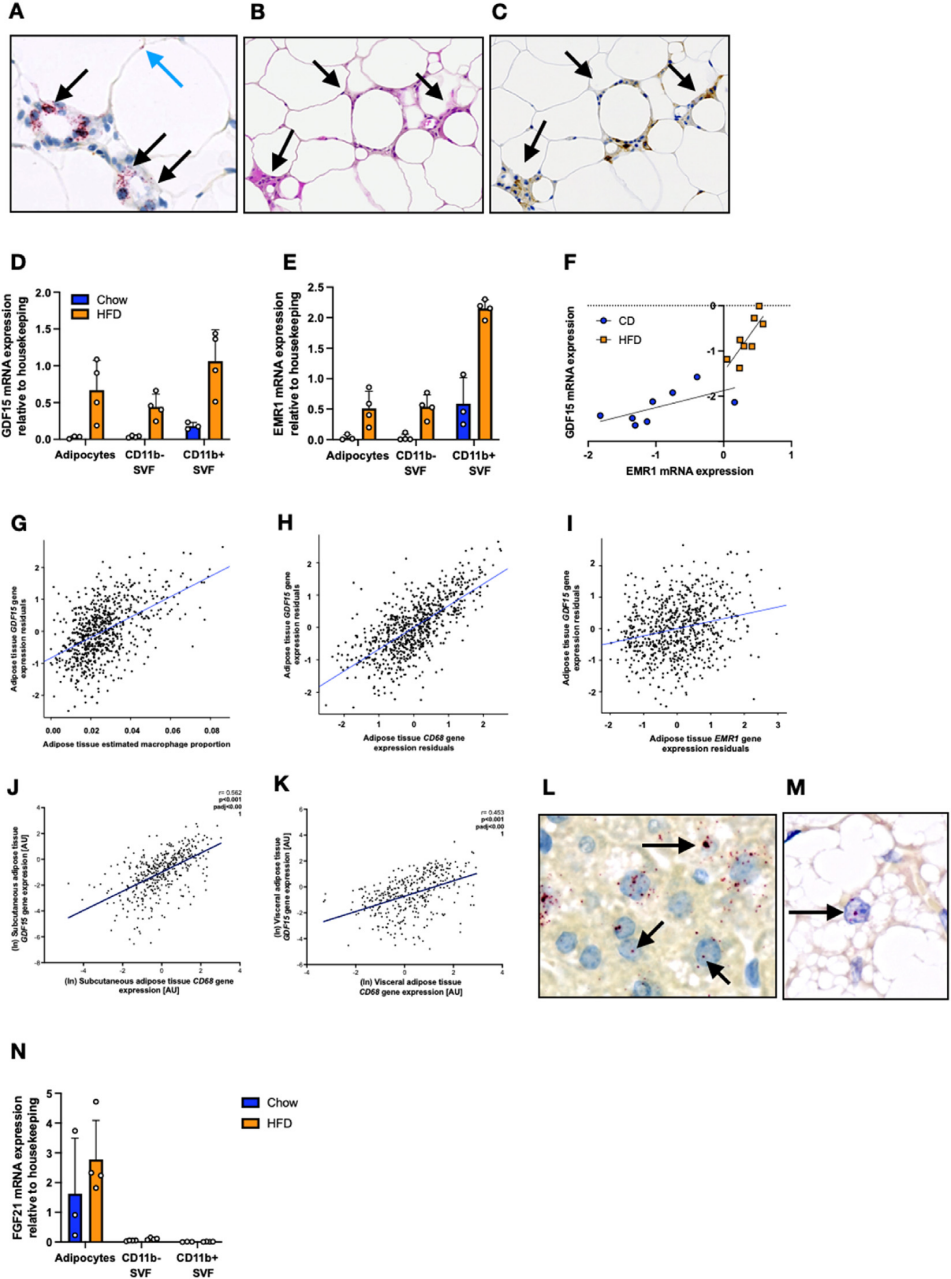
**GDF15 mRNA expression within highfat dietfed mouse and human adipose tissue**. (A) In situ hybridization analysis of GDF15 mRNA (red) from 18-week-old high fat diet (HFD) fed wild type (WT) mouse epididymal adipose tissue. Black arrows indicate GDF15 staining in foamy macrophages contributing to the formation of ‘crown-like structures’. Blue arrow indicates GDF15 staining within adipocytes. (B) Representative haematoxylin/eosin image of epididymal tissue from 18-week old HFD fed WT mouse. Black arrows indicate infiltrating cells. (C) Representative image (corresponding to the image in B) of epididymal tissue from 18-week-old HFD fed WT mouse stained with the macrophage marker F4/80 confirming that the cells contributing to the ‘crown like structures’ are macrophages. (D and E) GDF15 and EMR1 mRNA expression from 14 week old Chow or HFD fed WT mouse epididymal tissue fractionated into adipocytes, CD11b negative (−) and CD11b positive (+) stromal vascular fractions (SVF) (n = 3–4). (F) Correlation of GDF15 expression with EMR1 expression in epididymal tissue from 14 week Chow or HFD fed wild type mice (n = 8). (G–I) Human subcutaneous adipose tissue GDF15 gene expression levels in the TwinsUK adipose study associated with estimated macrophage proportion in adipose tissue (G), and macrophage markers CD68 and EMR1 (H and I). Each point represents data from a single individual. Plotted gene expression residuals of GDF15, CD68 and EMR1 were adjusted for age, BMI and RNA-Seq technical covariates. (J and K) GDF15 gene expression levels in the ‘Obese study’ associated with macrophage marker CD68 in human subcutaneous and visceral adipose tissue. (L and M) In situ hybridization analysis of Gdf15 mRNA (red) from 18-week old HFD fed WT mouse liver and brown adipose tissue. (N) FGF21 mRNA expression from 14 week Chow or HFD fed WT mouse epididymal tissue fractionated in to adipocytes, CD11b negative (−) and CD11b positive (+) stromal vascular fraction (SVF) (n = 3–4).

Having previously shown that high fat feeding in mice is associated with elevated plasma GDF15 levels and increased GDF15 mRNA in white and brown adipose tissue, and the liver, we used RNA scope to identify the cellular source. In white adipose tissue, the vast majority of GDF15 expression was detected within macrophages contributing to the formation of ‘crown-like structures’, along with occasional spots which appeared to be in adipocytes (Figure 2A–C). In chow fed mice, RT-PCR quantification confirmed that GDF15 mRNA was mainly expressed in myeloid cells (CD11b + SVF) with very little expression in adipocytes or CD11b- SVF (stromal vascular fraction) (Figure 2D). In HFD fed mice, GDF15 was readily detectable within all three eWAT fractions. However, GDF15 expression within the CD11b- SVF and adipocyte fractions is likely due to macrophage contamination, as they express the macrophage marker EMR1 which correlates with GDF15 expression (Figure 2E,F).

In keeping with these murine data, GDF15 mRNA expression in human subcutaneous adipose tissue samples in 733 individuals from the TwinsUK cohort was strongly associated with the estimated adipose tissue macrophage proportion (β = 32.81; *P* < 2.2 × 10^−16^) (Figure 2G), as well as with expression of macrophage markers CD68 (β = 0.68; *P* < 2.2 × 10^−16^) and EMR1 (β = 0.21; *P* = 3.5 × 10^−9^) (Figure 2H,I). We confirmed these findings in a second large obese cohort (n = 525) in which we also had access to paired visceral adipose tissue samples. In this sample set, GDF15 mRNA expression was again strongly correlated with CD68 mRNA in subcutaneous fat. The correlation was also present in visceral fat, though interestingly was slightly weaker in that depot (Subcutaneous fat: r = 0.562, *P <* 0.001; Visceral fat: r = 0.453, *P <* 0.001). Both associations withstand adjustment for age, sex and BMI (*Padj* < 0.001) (Figure 2J,K).

In liver samples from HFD fed mice, the RNA scope analysis suggested that GDF15 mRNA was primarily detectable in hepatocytes with no visually apparent contribution from non-hepatocyte cell populations (Figure 2L). Previously published work has suggested that GDF15 expression is also increased in human liver tissue samples in the context of NAFLD [106]. In brown adipose tissue, the GDF15 mRNA signal appeared to originate predominantly from selected brown adipocytes (Figure 2M). In contrast, HFD-induced FGF21 is reported to be predominantly expressed within adipocytes themselves whereas within the liver it is also expressed in hepatocytes [60]. We confirmed the former using RT-PCR in epididymal WAT fractions (Figure 2N).

### 3.3 Characterisation of macrophage specific GDF15 KO mouse models

**Figure 3:**
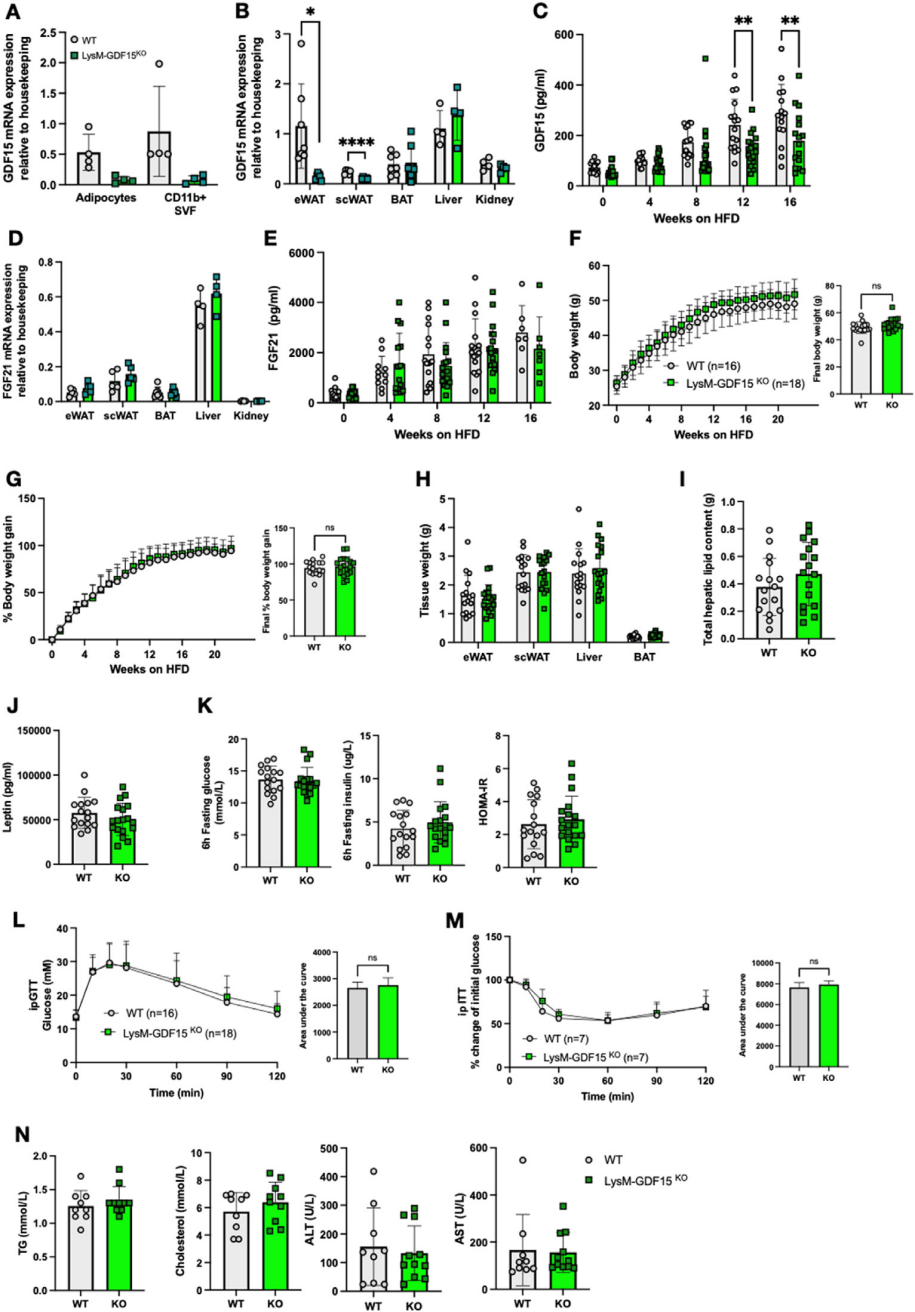
**Phenotypic characterization of LysM-Cre mediated GDF15 macrophage knockout mouse on a high fat diet (HFD)**. (A) GDF15 mRNA expression from 24-week-old high fat diet (HFD) fed wild type (WT) and LysM-GDF15^KO^ mouse epididymal adipose tissue fractionated into adipocytes and CD11b positive (+) stromal vascular fraction (SVF) (n = 4). (B) GDF15 mRNA expression in tissues from 24 week HFD fed WT and LysM-GDF15^KO^ mice. Epididymal white adipose tissue (eWAT), subcutaneous white adipose tissue (scWAT), brown adipose tissue (BAT), liver and kidney (n = 4–7). (C) Plasma GDF15 levels from random fed mice at indicated weeks on HFD (n = 15–18). (D) FGF21 mRNA expression in tissues from 24-week HFD fed WT and LysM-GDF15^KO^ mice (n = 4–7). (E) Plasma FGF21 levels from random fed mice at indicated weeks on HFD (n = 11–18). (F and G) Body weight and percent body weight gain of WT and LysM-GDF15^KO^ mice fed a 60% HFD; Inset, final body weight and percent body weight gain. (H) Weight of epididymal white adipose tissue (eWAT), subcutaneous white adipose tissue (scWAT), liver and brown adipose tissue (BAT) harvested at the end of the study, 24 weeks of HFD (n = 16–18). (I) Weight of total hepatic lipids in g; total lipid extracted from 25 mg tissue was normalized to total liver weight (n = 16,17). (J) Plasma leptin levels in mice from random fed mice after 16 weeks HFD-feeding (n = 16–18). (K) Blood glucose, plasma insulin and HOMA-IR levels from 6 h fasted mice, after 16 weeks of HFD feeding (n = 16,18). (L and M) Blood glucose levels during ip glucose tolerance test (GTT) and percent change from initial blood glucose levels during insulin tolerance test (ITT) after 16 weeks of HFD feeding. Inset, area under the curve analysis of glucose over time. (N) Plasma triglycerides (TG), cholesterol, alanine transaminase (ALT) and aspartate transaminase (AST) from random fed mice, after 16 weeks of HFD feeding (n = 9–11). All data are means ± S.D ∗/∗∗ - p < 0.05/0.01.

As macrophages appear to be the predominant site of GDF15 expression in adipose tissue, we proceeded to generate myeloid-specific GDF15 knockout mice (KO) using Cre recombinase under the control of the Lysozyme M promoter (LysM Cre). LysM-GDF15^KO^ were viable, fertile and born with no obvious abnormalities. Deletion of GDF15 was confirmed within isolated bone marrow derived macrophages (BMDMs), where we observed a >90% reduction in GDF15 mRNA (Supplemental Fig. 3). In keeping with these data, secretion of GDF15 into the media from isolated GDF15-null BMDMs was undetectable (Supplemental Figure 3). To verify that GDF15 was effectively deleted within the myeloid population *in vivo*, adipose tissue macrophages (ATMs) were purified from WT and LysM-GDF15^KO^ epididymal adipose tissue. Like in BMDMs, mRNA analysis revealed >90% reduction of GDF15 expression in the LysM-GDF15^KO^ ATMs (Figure 3A). Furthermore, analysis of gene expression from WAT displayed significant loss of GDF15 expression in LysM-GDF15^KO^ mice but not in other tissues (Figure 3B).

As expected, plasma concentrations of GDF15 increased during HFD feeding in all mice, but GDF15 concentrations were ∼30% lower in the LysM-GDF15^KO^ cohort (Figure 3C). FGF21 gene expression and plasma concentrations were similar between genotypes (Figure 3D,E). Weekly weight analysis revealed no differences in body weight or percent body weight gain in the LysM-GDF15^KO^ mice on a HFD compared to their WT counterparts (Figure 3F,G). Tissue weights, hepatic lipid content and plasma leptin were similar to WT mice (Figure 3H–J) as were fasting glucose, insulin, HOMA IR and both glucose and insulin tolerance (Figure 3K–M)**.** Plasma lipids (TG and cholesterol) were similar, as were plasma liver enzyme concentrations (Figure 3N).

**Figure 4:**
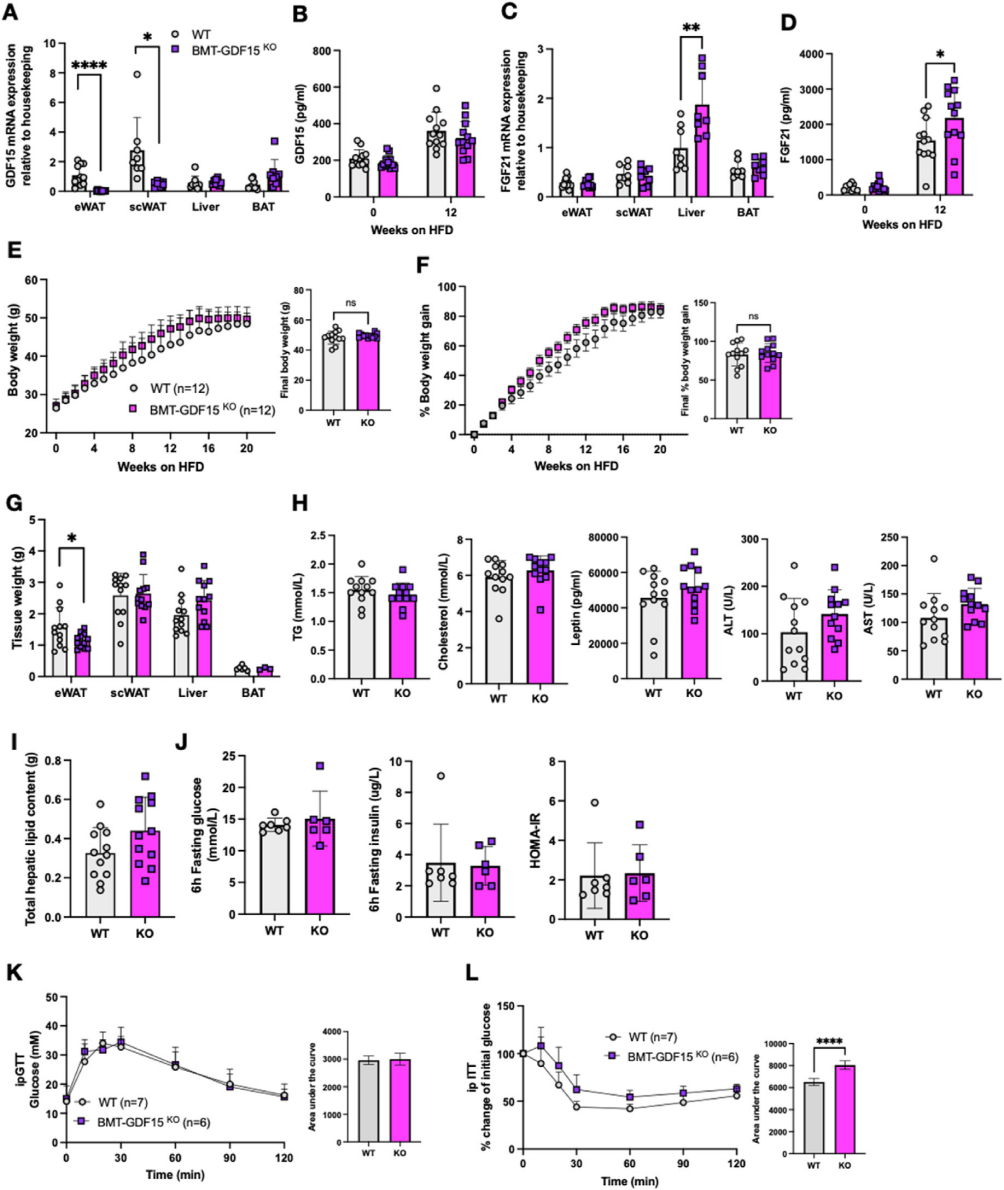
**Phenotypic characterization of bone marrow deleted GDF15 knockout mouse on a high fat diet**. (A) GDF15 mRNA expression in tissues from 24-week old high fat diet (HFD) fed WT and BMT-GDF15^KO^ mice. Epididymal white adipose tissue (eWAT), subcutaneous white adipose tissue (scWAT) and brown adipose tissue (BAT) (n = 8–12). (B) Plasma GDF15 levels from random fed mice at the onset (week 0) and after 12 weeks of HFD feeding (n = 12). (C) FGF21 mRNA expression from indicated tissues of 24 week HFD fed WT and BMT-GDF15^KO^ mice (n = 8–12). (D) Plasma FGF21 levels from random fed mice at the onset (week 0) and after 12 weeks of HFD feeding (n = 12). (E and F) Body weight and percent body weight gain of WT and BMT-GDF15^KO^ mice fed a 60% HFD; Inset, final body weight and percent body weight gain. (G) Weight of epididymal white adipose tissue (eWAT), subcutaneous white adipose tissue (scWAT), liver and brown adipose tissue (BAT) harvested at the end of the study, 24 weeks of HFD (n = 3–12). (H) Plasma triglycerides (TG), cholesterol, leptin, alanine transaminase (ALT) and aspartate transaminase (AST) from random fed mice, after 16 weeks of HFD feeding (n = 12). (I) Weight of total hepatic lipids in g; total lipid extracted from 25 mg tissue was normalized to total liver weight (n = 12). (J) Blood glucose, plasma insulin and HOMA-IR levels from 6 h fasted mice after 16 weeks of HFD feeding (n = 7,6). (K and L) Blood glucose levels during ip glucose tolerance test (GTT) and percent change from initial blood glucose levels during insulin tolerance test (ITT) after 16 weeks of HFD feeding. Inset, area under the curve analysis of glucose over time. All data are means ± S.D ∗/∗∗/∗∗∗/∗∗∗∗ - p < 0.05/0.01/0.001/0.0001.

Since the LysM-Cre driven deletion is present from birth and specifically targets the myeloid population, it is possible that compensatory mechanisms might account for the very modest impact on plasma GDF15 concentrations. To test this possibility, we transplanted bone marrow from GDF15 KO mice to regenerate the hematopoietic lineage in irradiated WT mice. GDF15 mRNA expression was effectively deleted within the BMT-GDF15^KO^ WAT depots but not in other tissues (liver and BAT) (Figure 4A). Within WAT, GDF15 mRNA was substantially reduced in the adipocyte fraction and in the CD11b - and CD11b + SVFs (Supplemental Figure 4). The fact that EMR1 mRNA expression was unchanged in these fractions in HF fed mice is consistent with macrophages being present in all 3 fractions as expected i.e. the macrophages are present but do not express GDF15 mRNA (Supplemental Figure 4A). In contrast, Plin1 mRNA was only present in the adipocyte fraction. We interpret these observations as being consistent with GDF15 mRNA in WAT largely being present in macrophages. Plasma GDF15 concentrations were similar in both lines (Figure 4B) and were substantially higher than in the LysM-Cre cohorts (Figure 3C). We presume that this relates to the irradiation therapy which is known to increase GDF15 [107,108]. However, similarly to what was observed in the whole body GDF15-null mice, plasma FGF21 concentrations and hepatic FGF21 mRNA expression were increased in the BMT-GDF15^KO^ mice (Figure 4C,D). When both groups were fed a 60% HFD, the BMT-GDF15^KO^ cohort displayed similar weight gain to the WT cohort (Figure 4E,F). Tissue weight analyses revealed a slight reduction in epididymal fat mass and a trend for increased liver weight (Figure 4G) but plasma TG, cholesterol, leptin concentrations as well as liver enzymes were similar (Figure 4H). Furthermore, there was no difference in hepatic fat content (Figure 4I). Fasting glucose and insulin levels, and HOMA IR were similar to WT levels as was glucose tolerance (Figure 4J,K), whereas insulin tolerance appeared to be modestly impaired (Figure 4L).

Collectively, these data suggest that myeloid cells are the main producer of adipose tissue GDF15 but contribute very little to the rise in circulating GDF15 concentrations seen in HFD fed mice. An implication of this finding is that white adipose tissue is likely to be a minor contributor to the rise in circulating GDF15 concentrations associated with weight gain. In keeping with this observation, plasma GDF15 concentrations were only weakly associated with white adipose tissue *GDF1*5 mRNA in the human TwinsUK cohort referred to previously (β = 0.08; *P* = 0.02) (Supplemental Figure 4B).

### 3.4 Characterisation of liver specific GDF15 null mice

**Figure 5:**
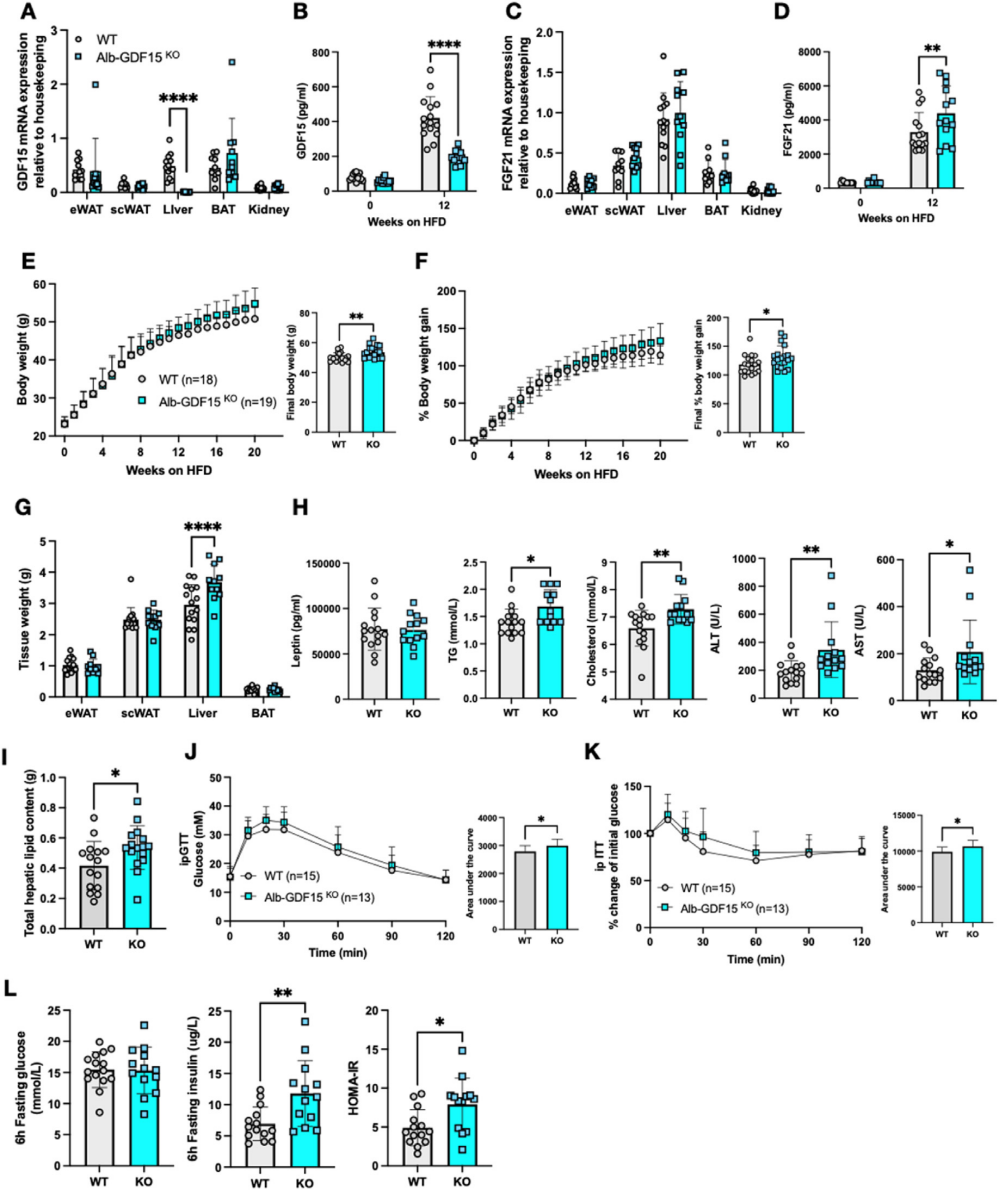
**Phenotypic characterization of Alb-Cre mediated GDF15 hepatocyte knockout mouse on a high fat diet**. (A) GDF15 mRNA expression in tissues from 24-week old high fat diet (HFD) fed wild type (WT) and Alb-GDF15^KO^ mice. Epididymal white adipose tissue (eWAT), subcutaneous white adipose tissue (scWAT), liver, brown adipose tissue (BAT) and kidney (n = 10–16). (B) Plasma GDF15 levels from random fed mice at the onset (week 0) and after 12 weeks of HFD feeding (n = 15,13). (C) FGF21 mRNA expression in indicated tissues from 24-week HFD fed WT and Alb-GDF15^KO^ mice (n = 10–13). (D) Plasma FGF21 levels from random fed mice at the onset (week 0) and after 12 weeks of HFD feeding (n = 15,13). (E and F) Body weight and percent body weight gain of WT and Alb-GDF15^KO^ mice fed a 60% HFD; Inset, final body weight and percent body weight gain. (G) Weight of epididymal white adipose tissue (eWAT), subcutaneous white adipose tissue (scWAT), liver and brown adipose tissue (BAT) harvested at the end of the study, 24 weeks of HFD (n = 15,11). (H) Plasma leptin, triglycerides (TG), cholesterol, alanine transaminase (ALT) and aspartate transaminase (AST) from random fed mice, after 16 weeks of HFD feeding (n = 15,13). (I) Weight of total hepatic lipids in g; total lipid extracted from 25 mg tissue was normalized to total liver weight (n = 15,16). (J and K) Blood glucose levels during ip glucose tolerance test (GTT) and percent change from initial blood glucose levels during insulin tolerance test (ITT) after 16 weeks of HFD feeding. Inset, area under the curve analysis of glucose over time. (L) Blood glucose, plasma insulin and HOMA-IR levels from 6 h fasted mice, after 16 weeks of HFD feeding (n = 14,13). All data are means ± S.D ∗/∗∗/∗∗∗/∗∗∗∗ - p < 0.05/0.01/0.001/0.0001.

In order to evaluate the contribution of hepatocyte derived GDF15, we used an Albumin driven Cre line (Alb-Cre) to generate hepatocyte-specific GDF15 null mice. Effective deletion of GDF15 was confirmed within Alb-GDF15^KO^ liver tissue where there was a 90% reduction in GDF15 mRNA expression without any changes in GDF15 mRNA in the other tissues examined (Figure 5A). Furthermore, in this instance plasma GDF15 was significantly reduced (∼50%) in HFD fed mice (Figure 5B), establishing the liver as a major source of circulating GDF15 in the HFD fed state. Alb-GDF15^KO^ mice also displayed elevated circulating FGF21 levels upon HFD feeding, but FGF21 mRNA expression in the liver was unchanged (Figure 5C,D). Alb-GDF15^KO^ mice were slightly heavier when fed a HFD compared to the WT control groups (Figure 5E,F) though leptin concentrations were similar to those of WT littermates (Figure 5H) and we were not able to detect significant differences in food intake or energy expenditure (Supplemental Figure. 5A and B). However, the Alb-GDF15^KO^ mice did also manifest larger livers (Figure 5G), higher plasma lipids (TG, cholesterol) and liver transaminases (ALT, AST) (Figure 5H). Histologically, Alb-GDF15^KO^ livers appeared to be similar to WT control mice (Supplemental Figure 5C), though total hepatic lipid content was significantly higher as the livers were heavier (Figure 5I). mRNA analysis revealed no difference in hepatic expression of genes involved in lipid synthesis or fat oxidation (Supplemental Figure 5D). Metabolically, Alb-GDF15^KO^ mice showed minor impairments in glucose and insulin tolerance as well as higher fasting insulin concentrations and thus higher HOMA IR scores (Figure 5J–L).

### 3.5 Characterisation of GDF15/FGF21 double KO mice

**Figure 6:**
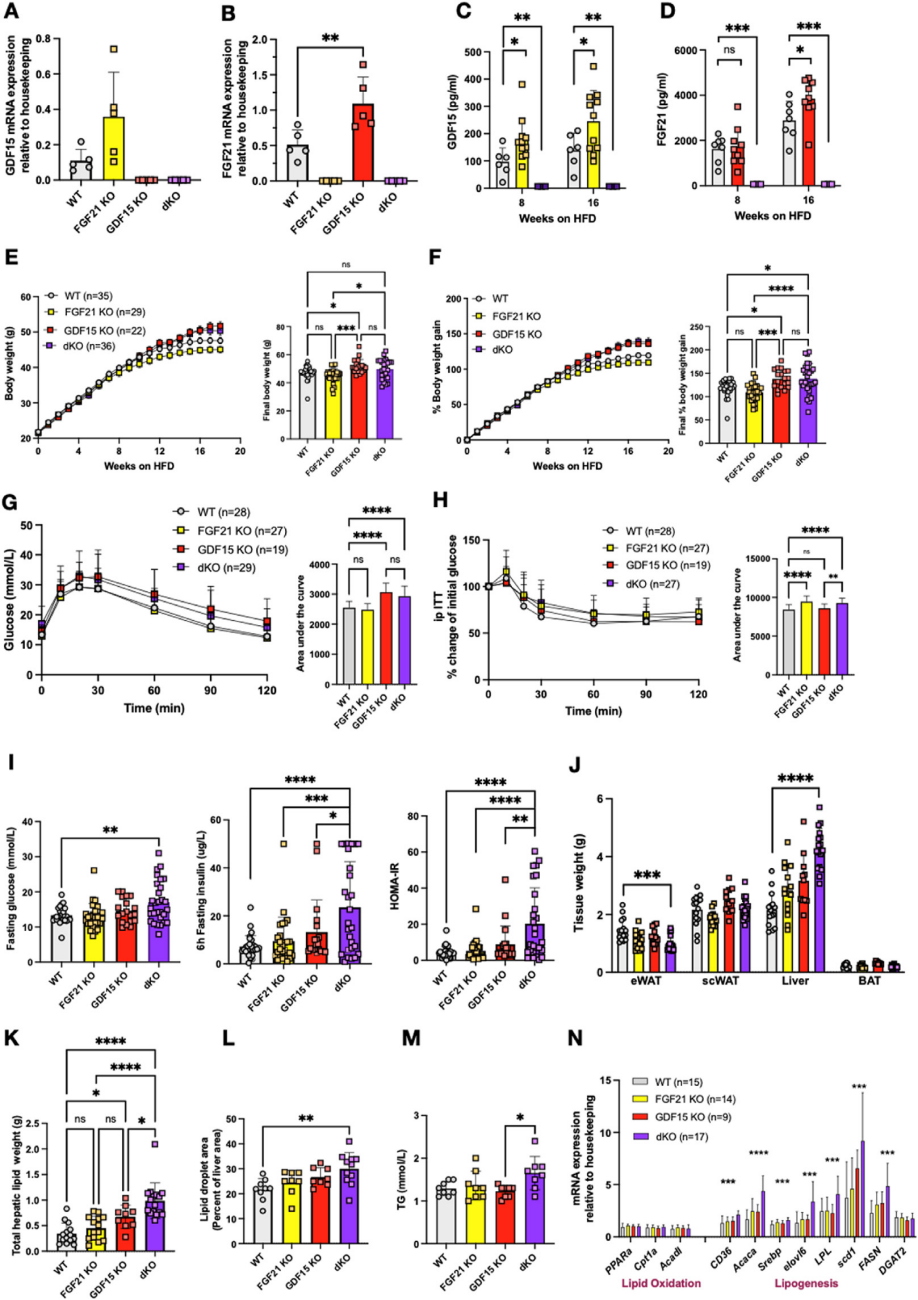
**Phenotypic characterization of dKO knockout mice on a high fat diet**. (A) Liver GDF15 and (B) FGF21 mRNA expression from 24-week-old high fat diet (HFD) fed WT, FGF21 KO, GDF15 KO and FGF21/GDF15 double knockout (dKO) mice (n = 5). (C and D) Plasma GDF15 and FGF21 from random fed mice at indicated time after high fat feeding (n = 3–11). (E and F) Body weight and percent body weight gain of WT, FGF21 KO, GDF15 KO and dKO mice fed a 60% HFD; Inset, final body weight and percent body weight gain. (G and H) Blood glucose levels during ip glucose tolerance test (GTT) and percent change from initial blood glucose levels during insulin tolerance test (ITT) after 16 weeks of HFD feeding. Inset, area under the curve analysis of glucose over time. (I) Blood glucose, plasma insulin and HOMA-IR levels from 6 h fasted mice after 16 weeks of HFD feeding (n = 19–29). (J) Weight of epididymal white adipose tissue (eWAT), subcutaneous white adipose tissue (scWAT), liver and brown adipose tissue (BAT) harvested at the end of the study, 24 weeks of HFD (n = 11–17). (K) Weight of total hepatic lipids; total lipid extracted from 25 mg tissue was normalized to total liver weight (n = 9–17). (L) Lipid droplet area (Percent liver area) determined from histological analyses of H&E stained liver sections (n = 8–11). (M) Plasma triglycerides (TG) from random fed mice, after 24 weeks of HFD feeding (n = 8). (N) Hepatic mRNA expression of genes involved in lipid metabolism (n = 9–17). All data are means ± S.D ∗/∗∗/∗∗∗/∗∗∗∗ - p < 0.05/0.01/0.001/0.0001. Please note that the data for WT and GDF15KO overlaps with the data presented in Figure 1.

Similarly to GDF15, circulating FGF21 has also been shown to be largely derived from the liver in obese HFD fed mice [60] raising the question of why two stress responsive hormones are secreted in this context of overnutrition. Furthermore, we had noted increased FGF21 concentrations in the plasma of GDF15 null mice suggesting that this might be attenuating the impact of GDF15 deficiency on HFD associated weight gain. In order to test this hypothesis we next studied mice lacking both GDF15 and FGF21 i.e. ‘double knockouts’. The GDF15/FGF21 double knockout (dKO) strain displayed normal viability and fertility and had no gross abnormalities. Loss of both hormones in dKO mice was confirmed by checking mRNA expression in the liver (Figure 6A,B) and circulating concentrations (Figure 6C,D). Interestingly, as seen for circulating FGF21, plasma GDF15 in FGF21KO mice was significantly higher than in wild-type littermates within 8 weeks of commencing a HFD diet and then further elevated after 16 weeks of HFD feeding (Figure 6C). dKO mice were slightly heavier than their WT littermates, but their weight was not significantly different from GDF15 KO mice (Figure 6E,F) and we did not detect significant differences in food intake or energy expenditure (Supplemental Figure 6A and B). Similarly to the GDF15 KOs, the dKO mice displayed mildly impaired glucose and insulin tolerance (as reflected by AUC) (Figure 6G,H). However, higher fasting glucose, insulin and thus HOMA IR scores suggested that they were significantly more insulin resistant than the GDF15 null mice, at least in the fasting state (Figure 6I). In contrast, FGF21 KO mice displayed relatively normal glucose tolerance and circulating insulin concentrations (Figure 6I).

Despite their body weight being similar to that of the single GDF15 KOs, the most prominent phenotype of the dKO mice was their increased liver size (Figure 6J). This was associated with increased hepatic lipid content and elevated circulating triglycerides (Figure 6K–M and Supplemental Figure 6C). Furthermore, plasma cholesterol, NEFA, AST and ALT levels were also significantly higher in the dKOs compared to the other genotypes (Supplemental Figure 6D). Gene expression analysis revealed increases in genes encoding proteins involved in *de novo* lipogenesis (DNL) whereas genes involved in fat oxidation were expressed at similar levels to the other genotypes studied (Figure 6N). This is likely to relate to the higher insulin concentrations in the dKO mice.

## 4 Discussion

GDF15 was identified more than 20 years ago as a gene that was upregulated in ‘activated macrophages’ but has since been shown to be elevated in a range of human disease states [55,80,109]. Our original interest in understanding GDF15 biology in the context of weight gain and metabolic disease related to three key observations: (i) GDF15 is elevated in human and rodent models of obesity [[42], [43], [44]]; (ii) transgenic overexpression or pharmacological treatment with GDF15, suppresses food intake and reduces body weight in mice [43,[84], [85], [86]]; and (iii) this effect is mediated exclusively via the hindbrain restricted expression of the GFRAL receptor [87,88]. Whilst significant progress has been made in understanding the mechanism by which GDF15 leads to suppression of food intake and modulation of body weight [84,87,88], the cellular source of GDF15 in this context remains unclear.

Here we first sought to determine (i) the source of circulating GDF15 in HFD fed mice and (ii) if selective deletion of this source replicates the more obese phenotype observed in global GDF15 KO mice on a HFD. We previously showed that expression of GDF15 mRNA is increased in white and brown adipose tissue and in the liver in mice fed a HFD for up to 18 weeks, whereas its expression in skeletal muscle in this context is very low and does not differ from that of chow fed mice [33]. We used RNA scope to reveal the cellular source of GDF15 in WAT to be almost exclusively macrophages and in the liver, to be the hepatocytes themselves. In brown fat, we see a weak signal in selected adipocytes as well as in stromal macrophages. In keeping with these murine observations, we observed a strong correlation between GDF15 mRNA and macrophage markers (CD68 and EMR1) in human white fat samples from two large independent cohorts. It is well established that the number of macrophages in white adipose tissue increases with weight gain so these findings could either simply relate to the higher number of macrophages present in the tissue and/or increased expression within individual macrophages.

Interestingly, despite this evidence suggesting that macrophages account for the increase in GDF15 mRNA in WAT associated with weight gain in humans and mice, our data does not suggest that macrophages contribute a great deal to circulating GDF15 concentrations in this context. This is borne out by the very modest impact of both LysM Cre driven GDF15 deletion in macrophages and the reconstitution of hematopoietic lineage cells from GDF15-null mice in irradiated wild type mice, on serum GDF15 levels in HFD fed mice. Furthermore, this and the hepatocyte specific deletion of GDF15, indicate that Kuppfer cells (liver resident macrophages) are not a major source of GDF15. In humans, we also noted that plasma GDF15 levels correlate weakly with WAT GDF15 expression.

When GDF15 was deleted in hepatocytes using the Alb-Cre promoter, we observed a more substantial impact on GDF15 concentrations in mice gaining weight on a HFD and an associated small increase in body weight, suggesting that the liver is likely to be the major source of GDF15 in this context. However, the data suggest that additional tissues probably also contribute small amounts towards circulating GDF15 concentrations in this paradigm. These data are also consistent with a recent human study which showed that plasma GDF15 levels correlated strongly with NAFLD progression [106]. We have previously shown that activation of the integrated stress response is likely to be mediating the increase in GDF15 mRNA expression in the liver, highlighting the ‘stress’ imposed on hepatocytes by overfeeding. Hepatocytes appear to be a major site of ectopic lipid accumulation when the capacity of white adipocytes to store surplus energy is overloaded, and these data suggest that this results in a stress response in the hepatocytes, one of the consequences of which is increased GDF15 secretion. In contrast to this stress response in hepatocytes, skeletal muscle does not appear to manifest a similar response in high fat fed mice. We are also not aware of activation of the ISR having been demonstrated in skeletal muscle in obese humans.

In terms of the impact of the changes in circulating GDF15 concentrations on body weight, we saw very modest changes which broadly correlated with the changes or lack thereof in GDF15 concentrations. This is consistent with the rather subtle impact of global GDF15 deficiency on body weight, and, in a fashion reminiscent of what has been reported for FGF21, contrasts with the more striking impact of ‘pharmacological’ administration of GDF15 [66,[76], [77], [78],^88^,[110], 111, [112]]. Interestingly we did observe higher FGF21 expression in the GDF15 null mice and in some of the tissue specific GDF15 knockout lines. In order to determine if this response might be mitigating the impact of GDF15 deficiency on body weight we proceeded to study mice deficient for both hormones. In this context, we also showed that circulating concentrations of GDF15 are higher in FGF21 deficient mice. Whilst body weight gain of the double KO (dKO) mice was slightly greater than that of wildtype littermates in response to HFD feeding, the weight of the dKO mice was no higher than that of GDF15 null mice (Figure 6C). We attribute our inability to detect significant changes in food intake or energy expenditure in the dKO mice to the very modest changes in body weight i.e. we would only anticipate very small changes in food intake or energy expenditure in this context. Recent work by Katsumura et al. [113] clearly showed that more substantial increases in hepatic and serum FGF21 and GDF15 induced in response to inhibition of CNOT6L deadenylase reduced food intake and increased energy expenditure, in keeping with prior work on their respective mechanisms of action [61,112].

Intriguingly, despite the dKO mice having very modestly increased body weight compared to WT controls and similar body weight to GDF15 KOs, they did manifest significantly higher fasting glucose, insulin and HOMA IR levels (5× fold) than wild-type mice and, both GDF15 and FGF21 ‘single knockouts’. However, the changes in glucose and insulin tolerance in these mice were very modest. We interpret these data as suggesting that loss of GDF15 and FGF21 primarily impacts the liver and thus hepatic rather than peripheral insulin sensitivity. In keeping with this suggestion, liver triglyceride concentrations were significantly higher in the dKO mice and this was accompanied by higher plasma triglycerides and liver transaminases. Some have speculated that GDF15 may directly, or more likely indirectly, impact hepatic fat metabolism [50,[114], [115], [116]] though exactly how it might do so remains to be elucidated and will require careful investigation. In this context, Kang et al. studied a model in which both GDF15 and FGF21 expression were induced in the liver in response to activation of the integrated stress response, though in this case deletion of a mitochondrial ribosome protein CRIF1 directly impaired mitochondrial function which has its own impacts on metabolism whilst also increasing serum concentrations of both GDF15 and FGF21. Their mechanistic studies led them to suggest that GDF15 was involved in regulating fat mass, hepatic lipid metabolism and energy expenditure whereas FGF21 was involved in regulating insulin sensitivity and glucose homeostasis (and in some instances EE and hepatic fat accumulation) [117,118].

Collectively the data indicate that similarly to the situation with FGF21, the liver is a major source of circulating GDF15 in the context of high fat feeding. The fact that Alb-Cre driven deletion of GDF15 does not entirely alleviate the HFD induced rise in GDF15 suggests that other tissues are also likely to be contributing smaller amounts. Importantly however, we have never seen an increase in GDF15 or FGF21 in skeletal muscle in the context of HF feeding despite other data suggesting that muscle can secrete both hormones in the face of mitochondrial stress for example [34,119,120]. GDF15 deficiency does appear to result in an increase in high fat feeding induced weight gain though the size of this effect is modest. We interpret this as reflecting the primary role of GDF15 as a stress responsive hormone which has evolved to reduce eating and promote rest in the context of more acute insults such as might occur after ingesting a toxin. FGF21 is also highly expressed in the liver in high fat fed mice and does appear to have an additive impact on alleviating fatty liver and preserving insulin sensitivity. Further work will be required to elucidate exactly how these effects are mediated. Despite the very modest impact of GDF15 and FGF21 ‘loss-of-function’ on body weight, they remain of considerable interest as pharmacological ‘gain-of-function’ targets in relation to their impact on body weight and on hepatic steatosis.

## Availability of data and materials

Further information and requests for resources and reagents should be directed to and will be fulfilled by the Lead Contact, David Savage (dbs23@medschl.cam.ac.uk).

## Funding

This work was supported by the 10.13039/501100000265Medical Research Council Metabolic Diseases Unit [MC_UU_00014/5] and the 10.13039/100010269Wellcome Trust Major Award [208363/Z/17/Z]. J.A.T is supported by the 10.13039/501100000265MRC Metabolic Diseases Unit (MC_UU_00014/1) and by a 10.13039/501100000272NIHR Clinical Lectureship (CL-2019-14-504). D.B.S (WT 219417) and S.O. are supported by the 10.13039/100010269Wellcome Trust (WT 214274/Z/18/Z), the 10.13039/501100000265MRC Metabolic Disease Unit (MC_UU_00014/1), and the 10.13039/501100018956NIHR Cambridge Biomedical Research Centre and 10.13039/501100000272NIHR Rare Disease Translational Research Collaboration. S.V. and G.B. are supported by The 10.13039/501100000274British Heart Foundation (RG/18/7/33636), and by the 10.13039/501100000265MRC (MC_UU_00014/2). M.B and E.G-J. was supported by the 10.13039/501100001659Deutsche Forschungsgemeinschaft through CRC 1052, project number 209933838, subproject B1 to M.B. and by Deutsches Zentrum für Diabetesforschung (DZD, Grant: 82DZD00601) to M.B and E.G-J. KSS acknowledges funding from the 10.13039/501100000265Medical Research Council (MR/M004422/1 and MR/R023131/1). TwinsUK is funded by the 10.13039/100010269Wellcome Trust, 10.13039/501100000265Medical Research Council, European Union, 10.13039/100011721Chronic Disease Research Foundation (CDRF), Zoe Global Ltd and the 10.13039/100017751National Institute for Health Research (NIHR) BioResource, Clinical Research Facility and 10.13039/100014461Biomedical Research Centre based at 10.13039/501100004941Guy’s and St Thomas’ NHS Foundation Trust in partnership with 10.13039/501100000764King’s College London.

## Data Availability

Data will be made available on request.

## References

[1] Arner P., Rydén M. (2022). Human white adipose tissue: a highly dynamic metabolic organ. Journal of Internal Medicine.

[2] Tremblay A., Després J., Thériault G., Fournier G., Bouchard C. (1992). Overfeeding expenditure in humans. The American Journal of Clinical Nutrition.

[3] Carpenter K.C., Strohacker K., McFarlin B.K. (2013). Considerations to maximize fat mass gain in a mouse model of diet-induced weight gain. Laboratory Animals.

[4] Lindhorst A., Raulien N., Wieghofer P., Eilers J., Rossi F.M.V., Bechmann I. (2021). Adipocyte death triggers a pro-inflammatory response and induces metabolic activation of resident macrophages. Cell Death & Disease.

[5] Cinti S. (2012). The adipose organ at a glance. DMM Disease Models and Mechanisms.

[6] Skurk T., Alberti-Huber C., Herder C., Hauner H. (2007). Relationship between adipocyte size and adipokine expression and secretion. Journal of Clinical Endocrinology and Metabolism.

[7] Weisberg S.P., McCann D., Desai M., Rosenbaum M., Leibel R.L., Ferrante A.W. (2003). Obesity is associated with macrophage accumulation in adipose tissue. Journal of Clinical Investigation.

[8] Virtue S., Vidal-Puig A. (2010). Adipose tissue expandability, lipotoxicity and the Metabolic Syndrome - an allostatic perspective. Biochimica et Biophysica Acta, Molecular and Cell Biology of Lipids.

[9] Murano I., Barbatelli G., Parisani V., Latini C., Muzzonigro G., Castellucci M. (2008). Dead adipocytes, detected as crown-like structures, are prevalent in visceral fat depots of genetically obese mice. Journal of Lipid Research.

[10] Savage D.B., Murgatroyd P.R., Chatterjee V.K., O'Rahilly S. (2005). Energy expenditure and adaptive responses to an acute hypercaloric fat load in humans with lipodystrophy. Journal of Clinical Endocrinology and Metabolism.

[11] Loomba R., Friedman S.L., Shulman G.I. (2021). Mechanisms and disease consequences of nonalcoholic fatty liver disease. Cell.

[12] Samuel V.T., Shulman G.I. (2018). Nonalcoholic fatty liver disease as a nexus of metabolic and hepatic diseases. Cell Metabolism.

[13] Roden M., Shulman G.I. (2019). The integrative biology of type 2 diabetes. Nature.

[14] Sanyal A.J., Campbell-Sargent C., Mirshahi F., Rizzo W.B., Contos M.J., Sterling R.K. (2001). Nonalcoholic steatohepatitis: association of insulin resistance and mitochondrial abnormalities. Gastroenterology.

[15] Kim J.K., Fillmore J.J., Chen Y., Yu C., Moore I.K., Pypaert M. (2001). Tissue-specific overexpression of lipoprotein lipase causes tissue-specific insulin resistance. Proceedings of the National Academy of Sciences of the United States of America.

[16] Marchesini G., Brizi M., Morselli-Labate A.M., Bianchi G., Bugianesi E., McCullough A.J. (1999). Association of nonalcoholic fatty liver disease with insulin resistance. The American Journal of Medicine.

[17] Korenblat K.M., Fabbrini E., Mohammed B.S., Klein S. (2008). Liver, muscle, and adipose tissue insulin action is directly related to intrahepatic triglyceride content in obese subjects. Gastroenterology.

[18] Loomba R., Wong R., Fraysse J., Shreay S., Li S., Harrison S. (2020). Nonalcoholic fatty liver disease progression rates to cirrhosis and progression of cirrhosis to decompensation and mortality: a real world analysis of Medicare data. Alimentary Pharmacology and Therapeutics.

[19] Huang D.Q., El-Serag H.B., Loomba R. (2021). Global epidemiology of NAFLD-related HCC: trends, predictions, risk factors and prevention. Nature Reviews Gastroenterology & Hepatology.

[20] Krssak M., Petersen K.F., Dresner A., DiPietro L., Vogel S.M., Rothman D.L. (1999). Erratum: intramyocellular lipid concentrations are correlated with insulin sensitivity in humans: a 1H NMR spectroscopy study (Rapid communication) (Diabetologia (1999) 42 (113-116)). Diabetologia.

[21] Pan D.A., Lillioja S., Kriketos A.D., Milner M.R., Baur L.A., Bogardus C. (1997). Skeletal muscle triglyceride levels are inversely related to insulin action. Diabetes.

[22] Petersen M.C., Shulman G.I. (2017). Roles of diacylglycerols and ceramides in hepatic insulin resistance. Trends in Pharmacological Sciences.

[23] Wu H., Ballantyne C.M. (2017). Skeletal muscle inflammation and insulin resistance in obesity. Journal of Clinical Investigation.

[24] Ozcan U., Cao Q., Yilmaz E., Lee A.-H., Iwakoshi N.N., Ozdelen E. (2016). Endoplasm. Encyclopedia of Parasitology.

[25] Hotamisligil G.S. (2010). Endoplasmic reticulum stress and the inflammatory basis of metabolic disease. Cell.

[26] Nakatani Y., Kaneto H., Kawamori D., Yoshiuchi K., Hatazaki M., Matsuoka T.A. (2005). Involvement of endoplasmic reticulum stress in insulin resistance and diabetes. Journal of Biological Chemistry.

[27] Gregor M.F., Yang L., Fabbrini E., Mohammed B.S., Eagon J.C., Hotamisligil G.S. (2009). Endoplasmic reticulum stress is reduced in tissues of obese subjects after weight loss. Diabetes.

[28] Koh H.J., Toyoda T., Didesch M.M., Lee M.Y., Sleeman M.W., Kulkarni R.N. (2013). Tribbles 3 mediates endoplasmic reticulum stress-induced insulin resistance in skeletal muscle. Nature Communications.

[29] Deldicque L., Van Proeyen K., Francaux M., Hespel P. (2011). The unfolded protein response in human skeletal muscle is not involved in the onset of glucose tolerance impairment induced by a fat-rich diet. European Journal of Applied Physiology.

[30] Costa-Mattioli M., Walter P. (2020). The integrated stress response: from mechanism to disease. Science (New York, N.Y.).

[31] Hotamisligil G.S., Davis R.J. (2016). Cell signaling and stress responses. Cold Spring Harbor Perspectives in Biology.

[32] Pakos-Zebrucka K., Koryga I., Mnich K., Ljujic M., Samali A., Gorman A.M. (2016). The integrated stress response. EMBO Reports.

[33] Patel S., Alvarez-Guaita A., Melvin A., Rimmington D., Dattilo A., Miedzybrodzka E.L. (2019). GDF15 provides an endocrine signal of nutritional stress in mice and humans. Cell Metabolism.

[34] Chung H.K., Ryu D., Kim K.S., Chang J.Y., Kim Y.K., Yi H.S. (2017). Growth differentiation factor 15 is a myomitokine governing systemic energy homeostasis. Journal of Cell Biology.

[35] Dong K., Li H., Zhang M., Jiang S., Chen S., Zhou J. (2015). Endoplasmic reticulum stress induces up-regulation of hepatic β-Klotho expression through ATF4 signaling pathway. Biochemical and Biophysical Research Communications.

[36] Laeger T., Henagan T.M., Albarado D.C., Redman L.M., Bray G.A., Noland R.C. (2014). FGF21 is an endocrine signal of protein restriction. Journal of Clinical Investigation.

[37] Gómez-Ambrosi J., Gallego-Escuredo J.M., Catalán V., Rodríguez A., Domingo P., Moncada R. (2017). FGF19 and FGF21 serum concentrations in human obesity and type 2 diabetes behave differently after diet- or surgically-induced weight loss. Clinical Nutrition.

[38] Kralisch S., Tönjes A., Krause K., Richter J., Lossner U., Kovacs P. (2013). Fibroblast growth factor-21 serum concentrations are associated with metabolic and hepatic markers in humans. Journal of Endocrinology.

[39] Zhang X., Yeung D.C.Y., Karpisek M., Stejskal D., Zhou Z.G., Liu F. (2008). Serum FGF21 levels are increased in obesity and are independently associated with the metabolic syndrome in humans. Diabetes.

[40] Reinehr T., Woelfle J., Wunsch R., Roth C.L. (2012). Fibroblast Growth Factor 21 (FGF-21) and its relation to obesity, metabolic syndrome, and nonalcoholic fatty liver in children: a longitudinal analysis. Journal of Clinical Endocrinology and Metabolism.

[41] Kharitonenkov A., Shiyanova T.L., Koester A., Ford A.M., Micanovic R., Galbreath E.J. (2005). FGF-21 as a novel metabolic regulator. Journal of Clinical Investigation.

[42] Carballo-Casla A., García-Esquinas E., Buño-Soto A., Struijk E.A., López-García E., Rodríguez-Artalejo F. (2022). Metabolic syndrome and growth differentiation factor 15 in older adults. GeroScience.

[43] Xiong Y., Walker K., Min X., Hale C., Tran T., Komorowski R. (2017). Long-acting MIC-1/GDF15 molecules to treat obesity: evidence from mice to monkeys. Science Translational Medicine.

[44] Vila G., Riedl M., Anderwald C., Resl M., Handisurya A., Clodi M. (2011). The relationship between insulin resistance and the cardiovascular biomarker growth differentiation factor-15 in obese patients. Clinical Chemistry.

[45] Chavez A.O., Molina-Carrion M., Abdul-Ghani M.A., Folli F., DeFronzo R.A., Tripathy D. (2009). Circulating fibroblast growth factor-21 is elevated in impaired glucose tolerance and type 2 diabetes and correlates with muscle and hepatic insulin resistance. Diabetes Care.

[46] Kempf T., Guba-Quint A., Torgerson J., Magnone M.C., Haefliger C., Bobadilla M. (2012). Growth differentiation factor 15 predicts future insulin resistance and impaired glucose control in obese nondiabetic individuals: results from the XENDOS trial. European Journal of Endocrinology.

[47] Tucker B., Li H., Long X., Rye K.A., Ong K.L. (2019). Fibroblast growth factor 21 in non-alcoholic fatty liver disease. Metabolism: Clinical and Experimental.

[48] Dushay J., Chui P.C., Gopalakrishnan G.S., Varela-Rey M., Crawley M., Fisher F.M. (2010). Increased fibroblast growth factor 21 in obesity and nonalcoholic fatty liver disease. Gastroenterology.

[49] Rusli F., Deelen J., Andriyani E., Boekschoten M.V., Lute C., Van Den Akker E.B. (2016). Fibroblast growth factor 21 reflects liver fat accumulation and dysregulation of signalling pathways in the liver of C57BL/6J mice. Scientific Reports.

[50] Kim K.H., Kim S.H., Han D.H., Jo Y.S., Lee Y.H., Lee M.S. (2018). Growth differentiation factor 15 ameliorates nonalcoholic steatohepatitis and related metabolic disorders in mice. Scientific Reports.

[51] Bilson J., Scorletti E., Bindels L.B., Afolabi P.R., Targher G., Calder P.C. (2021). Growth differentiation factor-15 and the association between type 2 diabetes and liver fibrosis in NAFLD. Nutrition & Diabetes.

[52] Galuppo B., Agazzi C., Pierpont B., Chick J., Li Z., Caprio S. (2022). Growth differentiation factor 15 (GDF15) is associated with non-alcoholic fatty liver disease (NAFLD) in youth with overweight or obesity. Nutrition & Diabetes.

[53] Suomalainen A., Elo J.M., Pietiläinen K.H., Hakonen A.H., Sevastianova K., Korpela M. (2011). FGF-21 as a biomarker for muscle-manifesting mitochondrial respiratory chain deficiencies: a diagnostic study. The Lancet Neurology.

[54] Poulsen N.S., Madsen K.L., Hornsyld T.M., Eisum A.S.V., Fornander F., Buch A.E. (2020). Growth and differentiation factor 15 as a biomarker for mitochondrial myopathy. Mitochondrion.

[55] Keipert S., Ost M. (2021). Stress-induced FGF21 and GDF15 in obesity and obesity resistance. Trends in Endocrinology and Metabolism.

[56] Nishimura T., Nakatake Y., Konishi M., Itoh N. (2000). Identification of a novel FGF, FGF-21, preferentially expressed in the liver. Biochimica et Biophysica Acta, Gene Structure and Expression.

[57] Fisher F.M., Maratos-Flier E. (2016). Understanding the physiology of FGF21. Annual Review of Physiology.

[58] Kliewer S.A., Mangelsdorf D.J. (2019). A dozen years of discovery: insights into the physiology and pharmacology of FGF21. Cell Metabolism.

[59] Flippo K.H., Potthoff M.J. (2021). Metabolic messengers: FGF21. Nature Metabolism.

[60] Markan K.R., Naber M.C., Ameka M.K., Anderegg M.D., Mangelsdorf D.J., Kliewer S.A. (2014). Circulating FGF21 is liver derived and enhances glucose uptake during refeeding and overfeeding. Diabetes.

[61] Owen B.M., Ding X., Morgan D.A., Coate K.C., Bookout A.L., Rahmouni K. (2014). FGF21 acts centrally to induce sympathetic nerve activity, energy expenditure, and weight loss. Cell Metabolism.

[62] Douris N., Stevanovic D.M., Fisher F.M., Cisu T.I., Chee M.J., Nguyen N.L. (2015). Central fibroblast growth factor 21 browns white fat via sympathetic action in male mice. Endocrinology.

[63] Fisher F.F., Kleiner S., Douris N., Fox E.C., Mepani R.J., Verdeguer F. (2012). FGF21 regulates PGC-1α and browning of white adipose tissues in adaptive thermogenesis. Genes & Development.

[64] Dutchak P.A., Katafuchi T., Bookout A.L., Choi J.H., Yu R.T., Mangelsdorf D.J. (2012). Fibroblast growth factor-21 regulates PPARγ activity and the antidiabetic actions of thiazolidinediones. Cell.

[65] Bunney P.E., Zink A.N., Holm A.A., Billington C.J., Kotz C.M. (2017). Orexin activation counteracts decreases in nonexercise activity thermogenesis (NEAT) caused by high-fat diet. Physiology and Behavior.

[66] Singhal G., Fisher F.M., Chee M.J., Tan T.G., El Ouaamari A., Adams A.C. (2016). Fibroblast growth factor 21 (FGF21) protects against high fat diet induced inflammation and islet hyperplasia in pancreas. PLoS One.

[67] Foltz I.N., Hu S., King C., Wu X., Yang C., Wang W. (2012). Treating diabetes and obesity with an FGF21-mimetic antibody activating the βKlotho/FGFR1c receptor complex. Science Translational Medicine.

[68] Ding X., Boney-Montoya J., Owen B.M., Bookout A.L., Coate K.C., Mangelsdorf D.J. (2012). βKslotho is required for fibroblast growth factor 21 effects on growth and metabolism. Cell Metabolism.

[69] Adams A.C., Cheng C.C., Coskun T., Kharitonenkov A. (2012). FGF21 requires βklotho to act in vivo. PLoS One.

[70] Wente W., Efanov A.M., Brenner M., Kharitonenkov A., Köster A., Sandusky G.E. (2006). Fibroblast growth factor-21 improves pancreatic β-cell function and survival by activation of extracellular signal-regulated kinase 1/2 and Akt signaling pathways. Diabetes.

[71] Coskun T., Bina H.A., Schneider M.A., Dunbar J.D., Hu C.C., Chen Y. (2008). Fibroblast growth factor 21 corrects obesity in mice. Endocrinology.

[72] Camporez J.P.G., Jornayvaz F.R., Petersen M.C., Pesta D., Guigni B.A., Serr J. (2013). Cellular mechanisms by which FGF21 improves insulin sensitivity in male mice. Endocrinology.

[73] Kharitonenkov A., Wroblewski V.J., Koester A., Chen Y.F., Clutinger C.K., Tigno X.T. (2007). The metabolic state of diabetic monkeys is regulated by fibroblast growth factor-21. Endocrinology.

[74] Talukdar S., Zhou Y., Li D., Rossulek M., Dong J., Somayaji V. (2016). A long-acting FGF21 molecule, PF-05231023, decreases body weight and improves lipid profile in non-human primates and type 2 diabetic subjects. Cell Metabolism.

[75] Gaich G., Chien J.Y., Fu H., Glass L.C., Deeg M.A., Holland W.L. (2013). The effects of LY2405319, an FGF21 Analog, in obese human subjects with type 2 diabetes. Cell Metabolism.

[76] Li H., Wu G., Fang Q., Zhang M., Hui X., Sheng B. (2018). Fibroblast growth factor 21 increases insulin sensitivity through specific expansion of subcutaneous fat. Nature Communications.

[77] Adams A.C., Coskun T., Cheng C.C., O'Farrell L.S., DuBois S.L., Kharitonenkov A. (2013). Fibroblast growth factor 21 is not required for the antidiabetic actions of the thiazoladinediones. Molecular Metabolism.

[78] Fisher F.M., Chui P.C., Antonellis P.J., Bina H.A., Kharitonenkov A., Flier J.S. (2010). Obesity is a fibroblast growth factor 21 (FGF21)-resistant state. Diabetes.

[79] Bootcov M., Bauskin A., Valenzuela S., Moore A., Bansal M., He X. (1997). MIC-1, a novel macrophage inhibitory cytokine, is a divergent member of the TGF-beta superfamily. Proceedings of the National Academy of Sciences of USA.

[80] Breit S.N., Brown D.A., Tsai V.W.W. (2021). The GDF15-GFRAL pathway in Health and metabolic disease: friend or foe?. Annual Review of Physiology.

[81] Lockhart S.M., Saudek V., O'Rahilly S. (2020). Gdf15: a hormone conveying somatic distress to the brain. Endocrine Reviews.

[82] Wischhusen J., Melero I., Fridman W.H. (2020). Growth/differentiation factor-15 (GDF-15): from biomarker to novel targetable immune checkpoint. Frontiers in Immunology.

[83] Tran T., Yang J., Gardner J., Xiong Y. (2018). GDF15 deficiency promotes high fat diet- induced obesity in mice. PLoS One.

[84] Macia L., Tsai V.W.W., Nguyen A.D., Johnen H., Kuffner T., Shi Y.C. (2012). Macrophage inhibitory cytokine 1 (MIC-1/GDF15) decreases food intake, body weight and improves glucose tolerance in mice on normal & obesogenic diets. PLoS One.

[85] Johnen H., Lin S., Kuffner T., Brown D.A., Tsai V.W.W., Bauskin A.R. (2007). Tumor-induced anorexia and weight loss are mediated by the TGF-β superfamily cytokine MIC-1. Nature Medicine.

[86] Chrysovergis K., Wang X., Kosak J., Lee S.H., Kim J.S., Foley J.F. (2014). NAG-1/GDF-15 prevents obesity by increasing thermogenesis, lipolysis and oxidative metabolism. International Journal of Obesity.

[87] Mullican S.E., Lin-Schmidt X., Chin C.N., Chavez J.A., Furman J.L., Armstrong A.A. (2017). GFRAL is the receptor for GDF15 and the ligand promotes weight loss in mice and nonhuman primates. Nature Medicine.

[88] Emmerson P.J., Wang F., Du Y., Liu Q., Pickard R.T., Gonciarz M.D. (2017). The metabolic effects of GDF15 are mediated by the orphan receptor GFRAL. Nature Medicine.

[89] Itoh N. (2007). The Fgf families in humans, mice, and zebrafish: their evolutional processes and roles in development, metabolism, and disease. Biological and Pharmaceutical Bulletin.

[90] Pereiro P., Librán-Pérez M., Figueras A., Novoa B. (2020). Conserved function of zebrafish (Danio rerio) Gdf15 as a sepsis tolerance mediator. Developmental & Comparative Immunology.

[91] Adriaenssens A.E., Biggs E.K., Darwish T., Tadross J., Sukthankar T., Girish M. (2019). Glucose-dependent insulinotropic polypeptide receptor-expressing cells in the hypothalamus regulate food intake. Cell Metabolism.

[92] Dermitzakis E.T. (2015). Gene-gene and gene-environment interactions detected by transcriptome sequence analysis in twins. Nature Genetics.

[93] Klöting N., Fasshauer M., Dietrich A., Kovacs P., Schön M.R., Kern M. (2010). Insulin-sensitive obesity. American Journal of Physiology - Endocrinology And Metabolism.

[94] Langhardt J., Flehmig G., Klöting N., Lehmann S., Ebert T., Kern M. (2018). Effects of weight loss on glutathione peroxidase 3 serum concentrations and adipose tissue expression in human obesity. Obesity Facts.

[95] Rolle-Kampczyk U., Gebauer S., Haange S.B., Schubert K., Kern M., Moulla Y. (2020). Accumulation of distinct persistent organic pollutants is associated with adipose tissue inflammation. Science of the Total Environment.

[96] American Diabetes Association (2014). Diagnosis and classification of diabetes mellitus. Diabetes Care.

[97] Dobin A., Davis C.A., Schlesinger F., Drenkow J., Zaleski C., Jha S. (2013). STAR: ultrafast universal RNA-seq aligner. Bioinformatics.

[98] Glastonbury C.A., Couto Alves A., El-Sayed Moustafa J.S., Small K.S. (2019). Cell-type heterogeneity in adipose tissue is associated with complex traits and reveals disease-relevant cell-specific eQTLs. The American Journal of Human Genetics.

[99] Delaneau O., Ongen H., Brown A.A., Fort A., Panousis N.I., Dermitzakis E.T. (2017). A complete tool set for molecular QTL discovery and analysis. Nature Communications.

[100] Frankish A., Diekhans M., Ferreira A.M., Johnson R., Jungreis I., Loveland J. (2019). GENCODE reference annotation for the human and mouse genomes. Nucleic Acids Research.

[101] Mardinoglu A., Heiker J.T., Gärtner D., Björnson E., Schön M.R., Flehmig G. (2015). Extensive weight loss reveals distinct gene expression changes in human subcutaneous and visceral adipose tissue. Scientific Reports.

[102] Gesta S., Blühet M., Yamamoto Y., Norris A.W., Berndt J., Kralisch S. (2006). Evidence for a role of developmental genes in the origin of obesity and body fat distribution. Proceedings of the National Academy of Sciences of the United States of America.

[103] Newman A.M., Liu C.L., Green M.R., Gentles A.J., Feng W., Xu Y. (2015). Robust enumeration of cell subsets from tissue expression profiles. Nature Methods.

[104] Bates D., Mächler M., Bolker B.M., Walker S.C. (2015). Fitting linear mixed-effects models using lme4. Journal of Statistical Software.

[105] R Development Core team (2013).

[106] Govaere O., Cockell S., Tiniakos D., Queen R., Younes R., Vacca M. (2020). Transcriptomic profiling across the nonalcoholic fatty liver disease spectrum reveals gene signatures for steatohepatitis and fibrosis. Science Translational Medicine.

[107] Moritake T., Fujita H., Yanagisawa M., Nakawatari M., Imadome K., Nakamura E. (2012). Strain-dependent damage in mouse lung after carbon ion irradiation. International Journal of Radiation Oncology, Biology, Physics.

[108] Okazaki R., Moon Y., Norimura T., Eling T. (2006). Ionizing radiation enhances the expression of the nonsteroidal anti-inflammatory Drug-Activated Gene (NAG1) by increasing the expression of TP53 in human colon cancer cells. Radiation Research.

[109] Wang D., Day E.A., Townsend L.K., Djordjevic D., Jørgensen S.B., Steinberg G.R. (2021). GDF15: emerging biology and therapeutic applications for obesity and cardiometabolic disease. Nature Reviews Endocrinology.

[110] Yang L., Chang C.C., Sun Z., Madsen D., Zhu H., Padkjær S.B. (2017). GFRAL is the receptor for GDF15 and is required for the anti-obesity effects of the ligand. Nature Medicine.

[111] Mullican S.E., Lin-Schmidt X., Chin C.N., Chavez J.A., Furman J.L., Armstrong A.A. (2017). GFRAL is the receptor for GDF15 and the ligand promotes weight loss in mice and nonhuman primates. Nature Medicine.

[112] Hsu J.Y., Crawley S., Chen M., Ayupova D.A., Lindhout D.A., Higbee J. (2017). Non-homeostatic body weight regulation through a brainstem-restricted receptor for GDF15. Nature.

[113] Katsumura S., Siddiqui N., Goldsmith M.R., Cheah J.H., Fujikawa T., Minegishi G. (2022). Deadenylase-dependent mRNA decay of GDF15 and FGF21 orchestrates food intake and energy expenditure. Cell Metabolism.

[114] Li D., Zhang H., Zhong Y. (2018). Hepatic GDF15 is regulated by CHOP of the unfolded protein response and alleviates NAFLD progression in obese mice. Biochemical and Biophysical Research Communications.

[115] Tsai V.W.W., Zhang H.P., Manandhar R., Schofield P., Christ D., Lee-Ng K.K.M. (2019). GDF15 mediates adiposity resistance through actions on GFRAL neurons in the hindbrain AP/NTS. International Journal of Obesity.

[116] Luan H.H., Wang A., Hilliard B.K., Carvalho F., Rosen C.E., Ahasic A.M. (2019). GDF15 is an inflammation-induced central mediator of tissue tolerance. Cell.

[117] Kang S.G., Choi M.J., Jung S.B., Chung H.K., Chang J.Y., Kim J.T. (2021). Differential roles of GDF15 and FGF21 in systemic metabolic adaptation to the mitochondrial integrated stress response. iScience.

[118] Choi M.J., Jung S.B., Lee S.E., Kang S.G., Lee J.H., Ryu M.J. (2020). An adipocyte-specific defect in oxidative phosphorylation increases systemic energy expenditure and protects against diet-induced obesity in mouse models. Diabetologia.

[119] Ost M., Igual Gil C., Coleman V., Keipert S., Efstathiou S., Vidic V. (2020). Muscle-derived GDF15 drives diurnal anorexia and systemic metabolic remodeling during mitochondrial stress. EMBO Reports.

[120] Forsström S., Jackson C.B., Carroll C.J., Kuronen M., Pirinen E., Pradhan S. (2019). Fibroblast growth factor 21 drives dynamics of local and systemic stress responses in mitochondrial myopathy with mtDNA deletions. Cell Metabolism.

