# Supplementary material for: Combined genetic deletion of GDF15 and FGF21 has modest effects on body weight, hepatic steatosis and insulin resistance in high fat fed mice
Source: Mol Metab. 2022 Sep 2;65:101589. doi: 10.1016/j.molmet.2022.101589 (PMC9486046; doi:10.1016/j.molmet.2022.101589)
Supplement: Multimedia component 1 [file mmc1.docx]

**
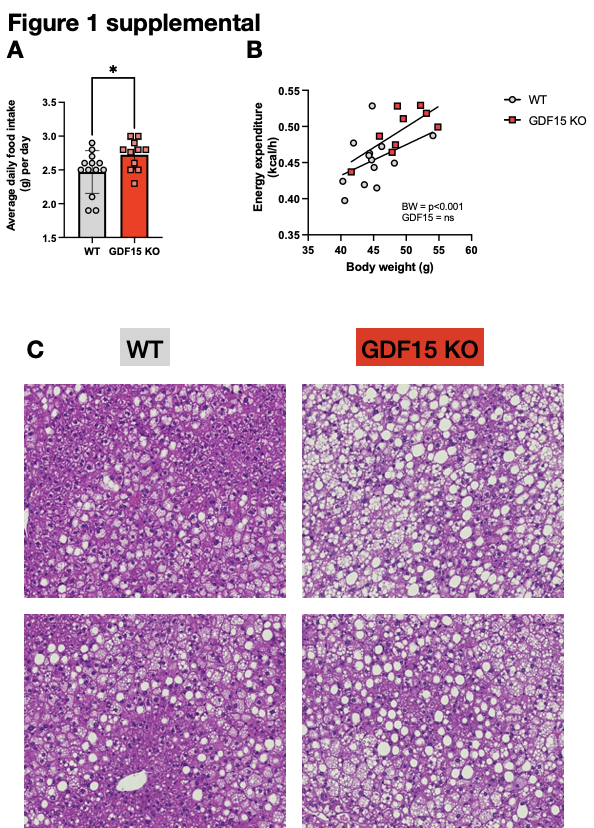
**

**Figure 1 Supplemental**

(A) Average daily food intake of WT and GDF15 KO after 22 weeks on a HFD, measured over a period of 2 weeks.

(B) Energy expenditure against body weight of mice. P values calculated using ANCOVA with average EE over 48 hours as the dependent variable, average body weight over 48 hours as a covariate and genotype as a fixed factor.

(C) Representative images of haematoxylin/eosin stained liver sections from WT and GDF15KO mice after 25 weeks HFD feeding.


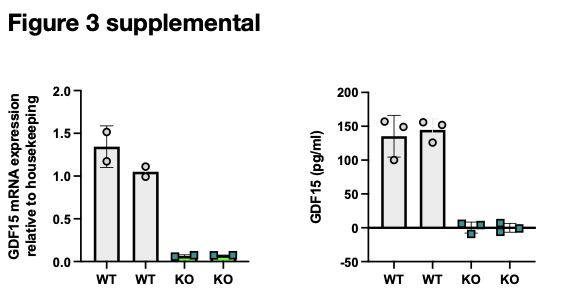


**Figure 3 Supplemental**

GDF15 mRNA expression and GDF15 secretion from WT and LysM-GDF15^KO^ bone-marrow derived macrophages (BMDM).

**
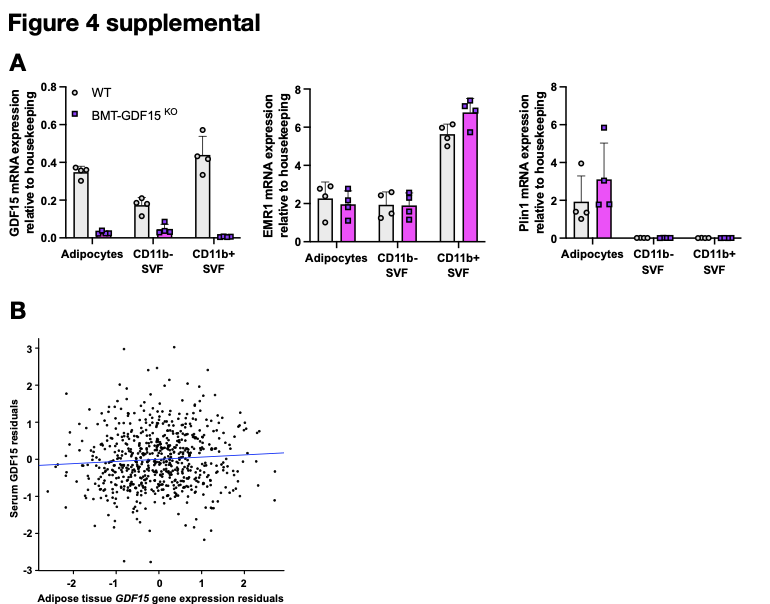
**

**Figure 4 Supplemental**

(A) GDF15, EMR1 and Plin1 mRNA expression from 24 week old high fat diet (HFD) fed wild type (WT) and BMT-GDF15^KO^ mouse epididymal adipose tissue fractionated into adipocytes and CD11b positive (+) stromal vascular fraction (SVF) (n=4).

(B) Human serum GDF15 levels show limited association with GDF15 gene expression levels in adipose tissue. Each point represents data from an individual. GDF15 serum levels were adjusted for age and BMI; plotted GDF15 gene expression residuals were adjusted for age, BMI and RNA-Seq technical covariates


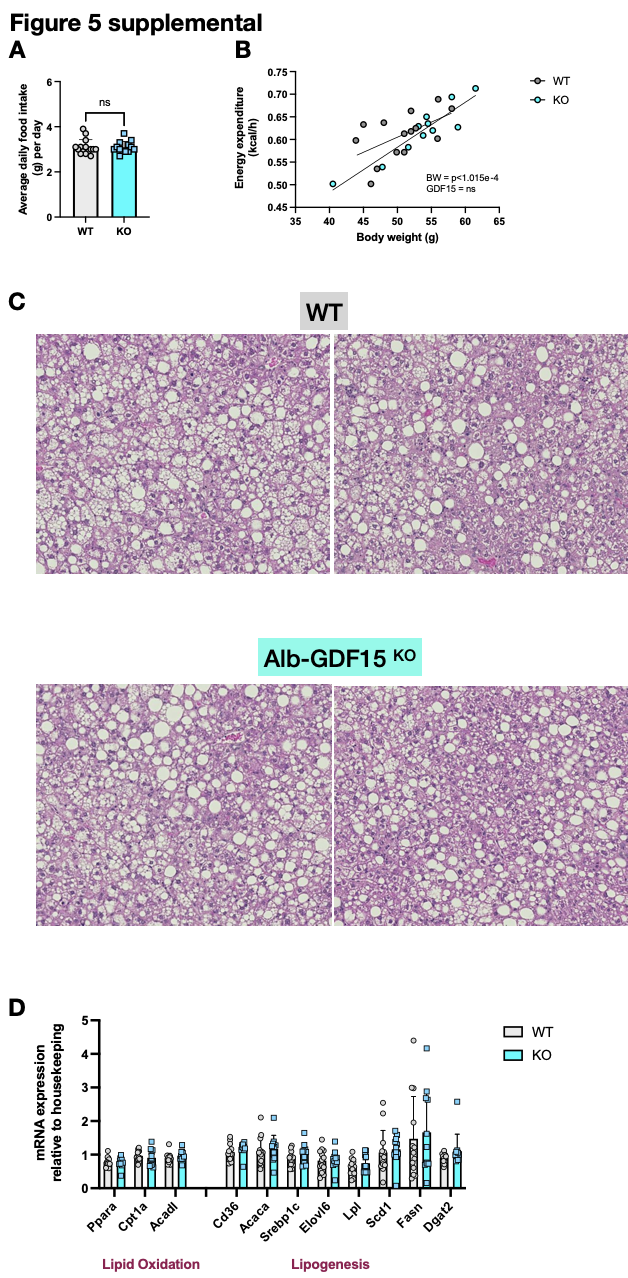


**Figure 5 Supplemental**

(A) Average daily food intake of WT and Alb-GDF15^KO^ on a HFD measured over a period of 2 weeks.

(B) Energy expenditure against body weight of mice. P values calculated using ANCOVA with average EE over 48 hours as the dependent variable, average body weight over 48 hours as a covariate and genotype as a fixed factor.

(C) Representative images of haematoxylin/eosin stained liver sections from WT and Alb-GDF15^KO^ mice after 24 weeks of HFD feeding

(D) Hepatic mRNA expression of genes involved in lipid metabolism (n=13,11).

**
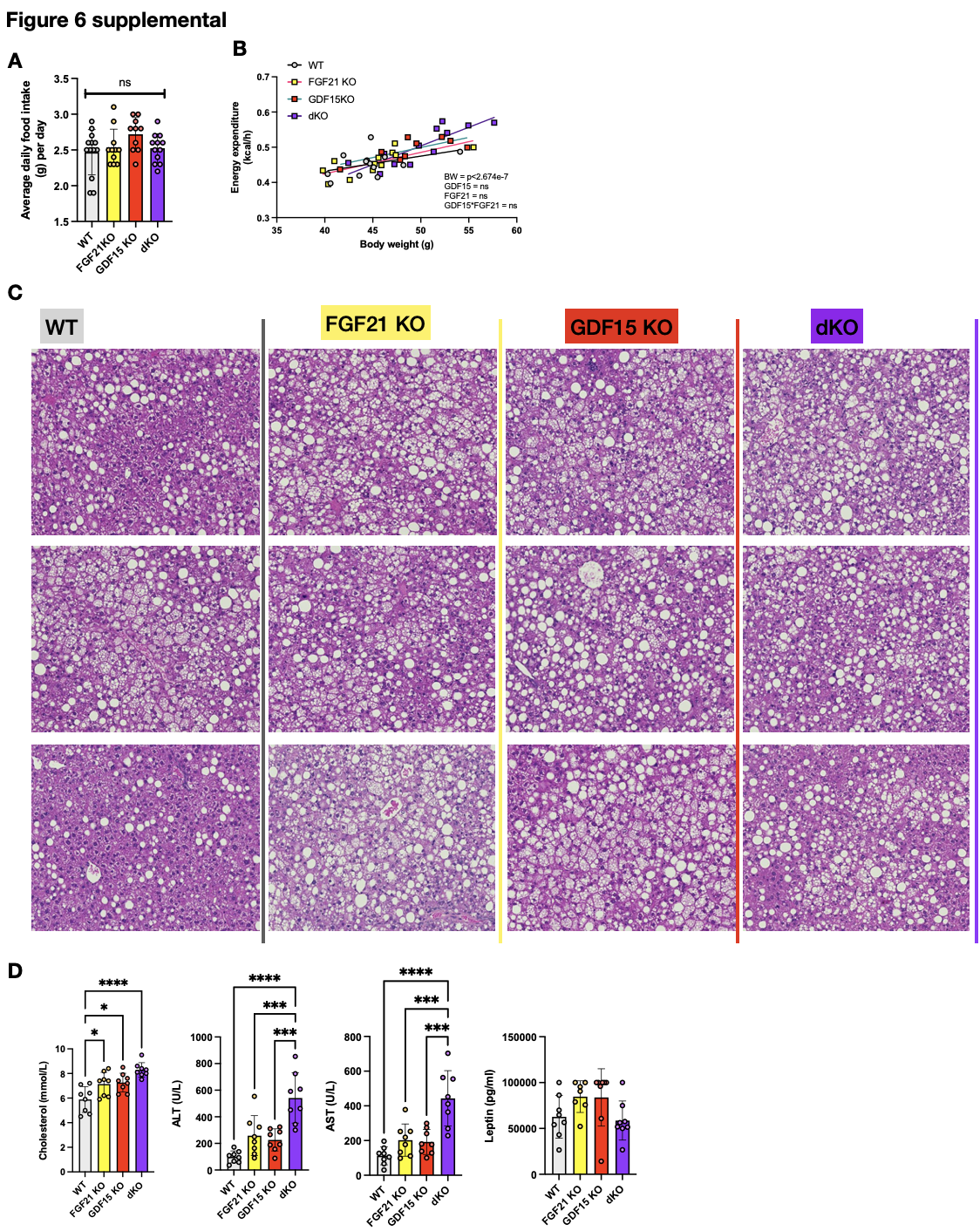
**

**Figure 6 Supplemental**

(A) Average daily food intake of WT, FGF21 KO, GDF15 KO and dKO after 22 weeks on a HFD measured over a period of 2 weeks

(B) Energy expenditure against body weight of mice. P values calculated using ANCOVA with average EE over 48 hours as the dependent variable, average body weight over 48 hours as a covariate and genotype as a fixed factor.

(C) Representative images of haematoxylin/eosin stained liver sections from WT, FGF21 KO, GDF15 KO and dKO mice after 25 weeks HFD feeding.

(D) Plasma cholesterol, alanine transaminase (ALT), aspartate transaminase (AST) and leptin from random fed mice, after 24 weeks of HFD-feeding (n=8).
